# Enantioselective cyclization reactions involving 3-isothiocyanato oxindoles: synthesis of spirocycles

**DOI:** 10.1039/d6ra01468b

**Published:** 2026-05-19

**Authors:** Mohammad Hadi Edareh, Fatemeh Doraghi, Arman Farahani, Mehran Ghasemi, Mohammad Mahdavi

**Affiliations:** a School of Chemistry, College of Science, University of Tehran Tehran Iran; b Endocrinology and Metabolism Research Center, Endocrinology and Metabolism Clinical Sciences Institute, Tehran University of Medical Sciences Tehran Iran momahdavi@tums.ac.ir; c Center for Research of Endemic Parasites of Iran, Tehran University of Medical Sciences Tehran Iran; d Natural and Medical Sciences Research Center (NMSRC), University of Nizwa Nizwa 616 Sultanate of Oman

## Abstract

Recently, 3-isothiocyanato oxindoles have been used in various asymmetric cascade cyclization reactions for the synthesis of biologically important spirocyclic compounds. As a powerful and versatile ambiphilic synthon, 3-isothiocyanato oxindoles react effectively with diverse electron-deficient unsaturated bonds through Michael addition/cyclization reactions under organocatalysis or metal catalysis systems. This review highlights catalytic enantioselective cyclization reaction of 3-isothiocyanato oxindoles since 2011, and discusses their mechanistic insights.

## Abstract 2

As a powerful ambiphilic synthon, 3-isothiocyanato oxindoles react effectively with diverse electron-deficient unsaturated bonds under organocatalysis or metal catalysis systems towards the synthesis of biologically important spirocyclic scaffolds.

## Graphical abstract

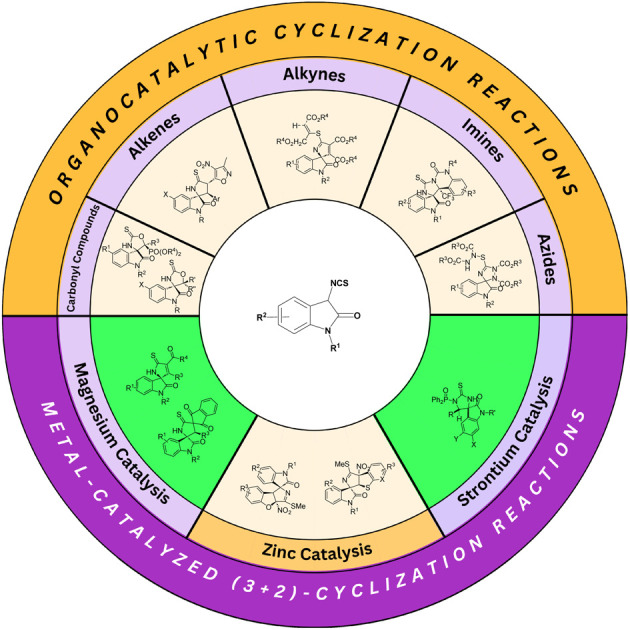

## 1. Introduction

**Fig. 1:**
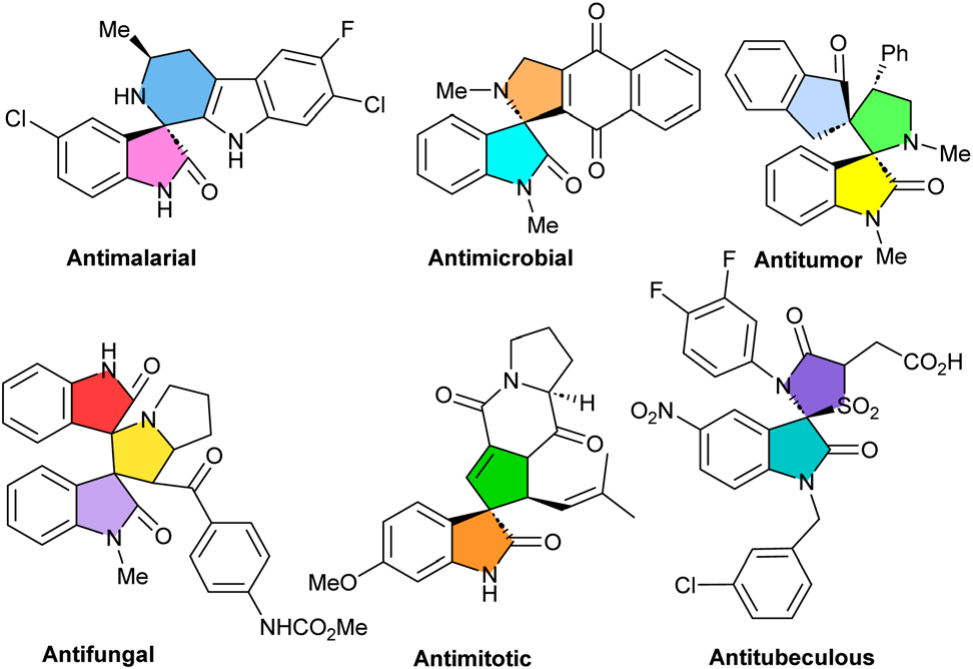
Some of the biologically active spirooxindoles.

Spirocyclic compounds have captured tremendous attention among synthetic and medicinal chemists, owing to their prevalence in numerous natural and unnatural products, as well as pharmaceutical compounds.^1–5^ Spirooxindole derivatives have significant biological properties such as antimalarial,^6^ antimicrobial,^7^ antitumor,^8^ antiviral,^9^ antifungal,^7^ antimitotic^10^ and antituberculosis^11^ activities. Some of these bioactive molecules are shown in Fig. 1.

3-Isothiocyanato oxindoles as powerful and versatile precursors can easily react with a diverse range of π-electrophiles through aldol/cyclization,^12^ Mannich/cyclization,^13^ and Michael/cyclization^14^ sequences to provide highly diastereo- and enantioselective synthesis of biologically important spirocyclic oxindoles. In 2011, Yuan and co-workers first used 3-isothiocyanato oxindoles in enantioselective (3 + 2)-cycloaddition reaction to make spirooxindole cores.^15^ Since their pioneering work, numerous spirooxindoles with a nitrogen atom at the C3′-position of the oxindole moiety have been synthesized, through the cyclization reaction of 3-isothiocyanato oxindoles with different electron-deficient reactants including alkenes, alkynes, carbonyl compounds, imines, and diazo compounds incorporate into such domino reactions. These asymmetric cyclization reactions can be catalyzed in the presence of a class of organocatalysts or metal catalysts to construct densely functionalized spirooxindoles containing multiple contiguous stereogenic centers.^16–19^

A variety of bifunctional organocatalysts, especially Cinchona alkaloid derived-squaramide or thiourea catalysts can catalyze reactions of 3-isothiocyanato oxindoles through forming hydrogen bonds to allow appropriate positions for two feedstocks, thus facilitate subsequent Michael addition and (3 + *n*)-cycloaddition. In the metal catalytic systems, metals like zinc, magnesium and strontium act as Lewis acids to coordinate with heteroatoms of two feedstocks.

The growing popularity of spirocyclic oxindoles as potential drugs has created demand for their enantioselective synthesis. In this review, we have specifically focused only on enantioselective cyclization reactions involving 3-isothiocyanato oxindoles. Hence, we have covered many enantioselective reactions under organometallic and metal catalytic systems since the first report of 3-isothiocyanato oxindoles under asymmetric conditions in 2011 to date, achieving a clear and logical comparison between these stereoselective environments and their mechanistic pathways.

## 2. Enantioselective cyclization reactions of 3-isothiocyanato oxindoles

### 2.1. Organocatalytic cyclization reactions of 3-isothiocyanato oxindoles

#### 2.1.1. Carbonyl compounds as electrophiles

**Scheme 1:**
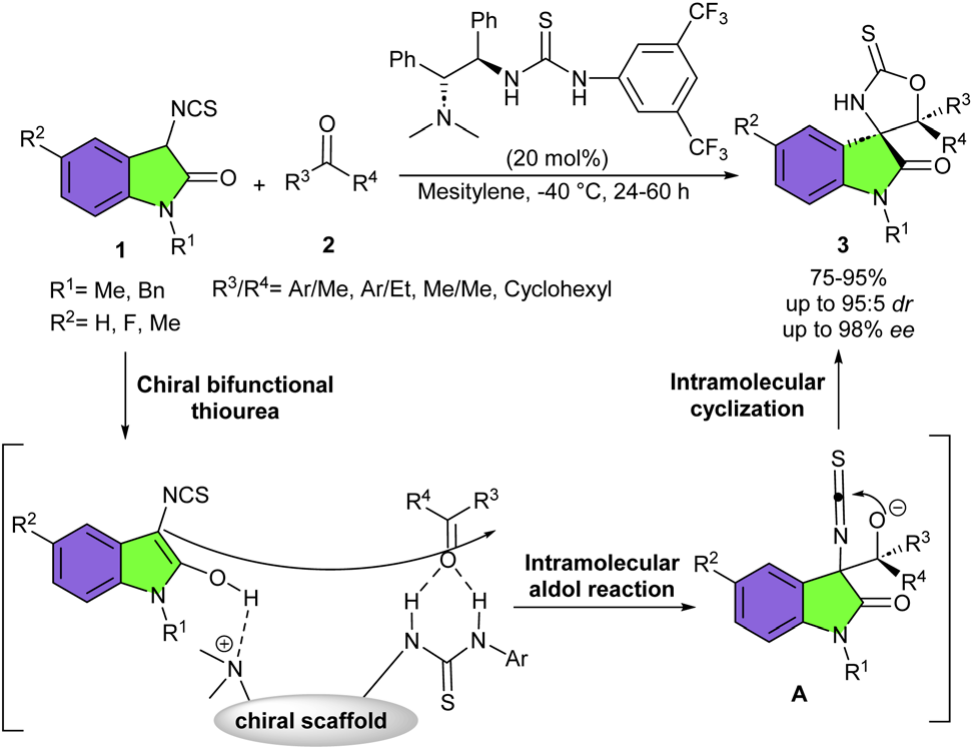
Organocatalytic reaction of 3-isothiocyanato oxindoles and ketones.

**Scheme 2:**
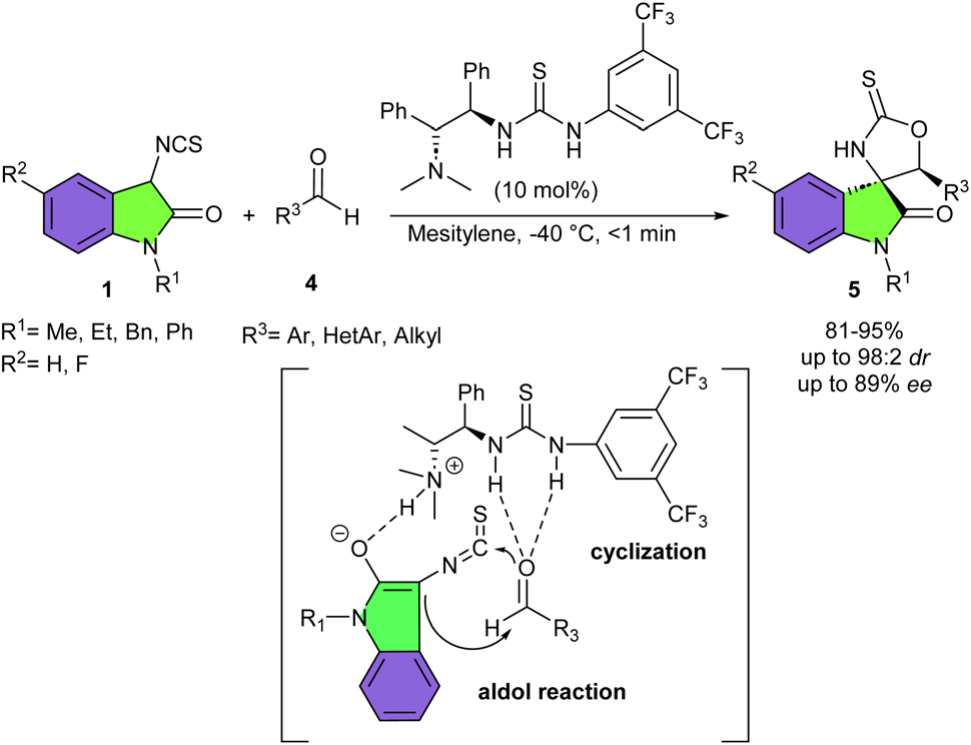
Organocatalytic reaction of 3-isothiocyanato oxindoles and aldehydes.

Pioneering work by the Yuan research team in 2011 demonstrated that 3-isothiocyanato oxindoles are highly reactive and efficient building blocks for the asymmetric synthesis of chiral spirooxindoles. They introduced a chiral bifunctional thiourea-tertiary amine organocatalyst in the reaction of 3-isothiocyanato oxindoles 1 and simple ketones 2 to synthesize a family of enantiomerically enriched spirooxindoles 3 containing two highly congested contiguous tetrasubstituted stereocenters (Scheme 1).^15^ The products were obtained in good to high yields (75–95%) with high enantio- and diastereoselectivities (up to 98% ee and up to 90 : 10 dr). Overall, the reaction mechanism involved the activation of both substrates 1 and 2*via* hydrogen-bonding interactions with the chiral bifunctional thiourea-tertiary amine. Then, an intramolecular aldol reaction occurred, generating intermediate A. This intermediate readily underwent subsequent intramolecular cyclization to afford the spirocyclic product 3. Several 3-isothiocyanato oxindole derivatives bearing neutral benzene ring or with F, or Me group showed good compatibilities. The incorporation of Me and Bn protecting groups on the nitrogen atom of 3-isothiocyanato oxindole did not affect the efficiency of the reaction. Acetophenones with either an electron-donating or electron-withdrawing group at the *meta* or *para* position of the aryl ring gave the corresponding products in good yield, good diastereoselectivity, and high enantioselectivity. While the *ortho*-substituted acetophenone and propiophenone did not workable. Excellent results were obtained by using 2-acetonaphthones. Aliphatic ketones also were amenable to this method, with high yields and moderate ee values. Finally, the authors were able to demonstrate the synthetic potential of this procedure using a large-scale experiment, which resulted in the production of 1.23 gr of product in 95% yield, with 94 : 6 dr, and 97% ee. In 2013, Yuan and his team used the same organocatalyst in the (3 + 2)-cyclization of 3-isothiocyanato oxindoles 1 with aldehydes 4 (Scheme 2).^12^ The procedure proceeded through cascade aldol-cyclization only within a few minutes. Aromatic, heteroaromatic and aliphatic aldehydes showed good reactivity with good diastereo- and enantioselectivities. Investigating of N–Et, N–Bn, and N–Ph 3-isothiocyanato also yielded the desired products in good yield and diastereoselectivity, but with moderate enantioselectivity, demonstrating the impact of the protecting group of N-1 on the stereoselectivity. Hence, the authors proposed a credible transition state in which the tertiary amine moiety of the organocatalyst deprotonated the active H-atom from 3-isothiocyanato oxindole tautomer and stabilized 3-isothiocyanato oxindole by an intermolecular hydrogen bonding interaction. At the same time, the thiourea moiety of the catalyst activated aldehyde *via* double hydrogen bonding to the O-atom of the aldehyde. So, the activated 3-isothiocyanato oxindole attacked the activated aldehyde through aldol reaction, and then, the O-atom attacked the NCS group of 3-isothiocyanato oxindole through cyclization reaction to obtain spiro[oxazolidine-2-thione-oxindole] products.

**Scheme 3:**
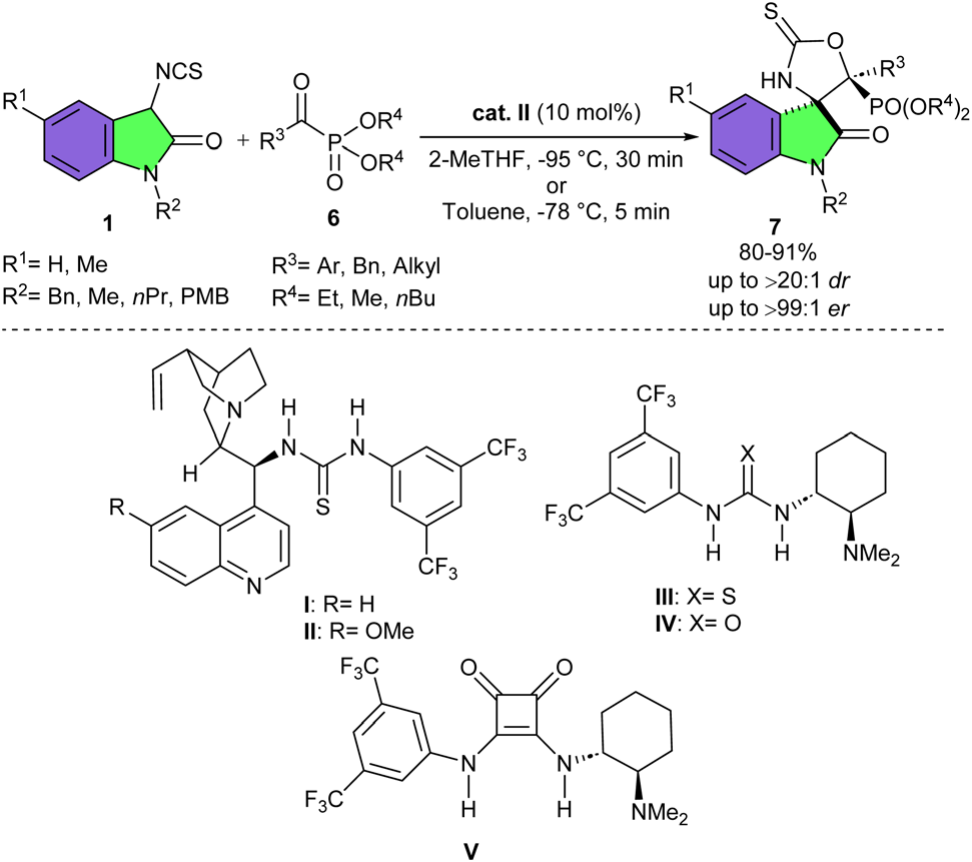
Quinine-based tertiary amino-thiourea-catalyzed reaction of 3-isothiocyanato oxindoles and α-ketophosphonates.

Screening of several organocatalysts in the aldol-cyclization reaction between 3-isothiocyanato oxindole 1 and α-ketophosphonate 6 in CH_2_Cl_2_ as a solvent at −78 °C for 5 min showed that organocatalysts I and II produced the desired product 7 in good diastereoselectivities and high enantioselectivities (10 : 1 dr 97 : 1 er, 15 : 1 dr and 99 : 1 er, respectively), while catalyst III gave the highest diastereoselevtivity (>20 : 1 dr), but lower enantioselectivity (6.5 : 93.5 er), catalysts IV and V led to lower stereoselectivities (5 : 1 dr, 3.5 : 96.5 er, and 7 : 1 dr, 4 : 96 er, respectively) (Scheme 3).^20^ Two reaction conditions were designed for the synthesis of β-amino-α-hydroxyphosphonate derivatives 7 in the presence of the optimal organocatalyst II. When the reaction conducted in 2-MeTHF as a solvent at −95 °C, both aliphatic and aromatic α-ketophosphonates reacted well with 3-isothiocyanato oxindoles. While aromatic α-ketophosphonates were good reactants in toluene as a solvent at −78 °C. The viability of this procedure was demonstrated by a large-scale experiment using only 2 mol% of the catalyst II, leading to 185 mg of the desired product in 80% yield, 10 : 1 dr, and 98 : 2 er.

#### 2.1.2. Alkenes as electrophiles

**Scheme 4:**
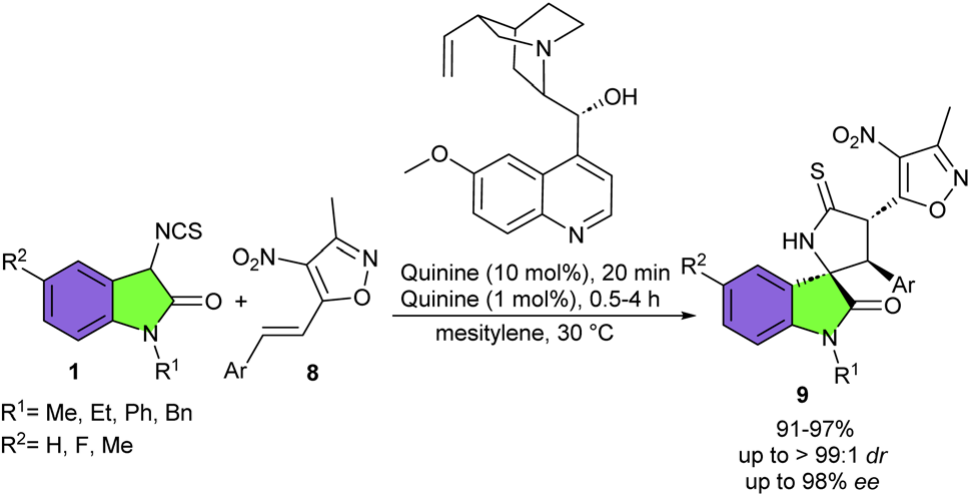
Quinine-catalyzed reaction of 3-isothiocyanato oxindoles and 3-methyl-4-nitro-5-alkenyl-isoxazoles.

**Scheme 5:**
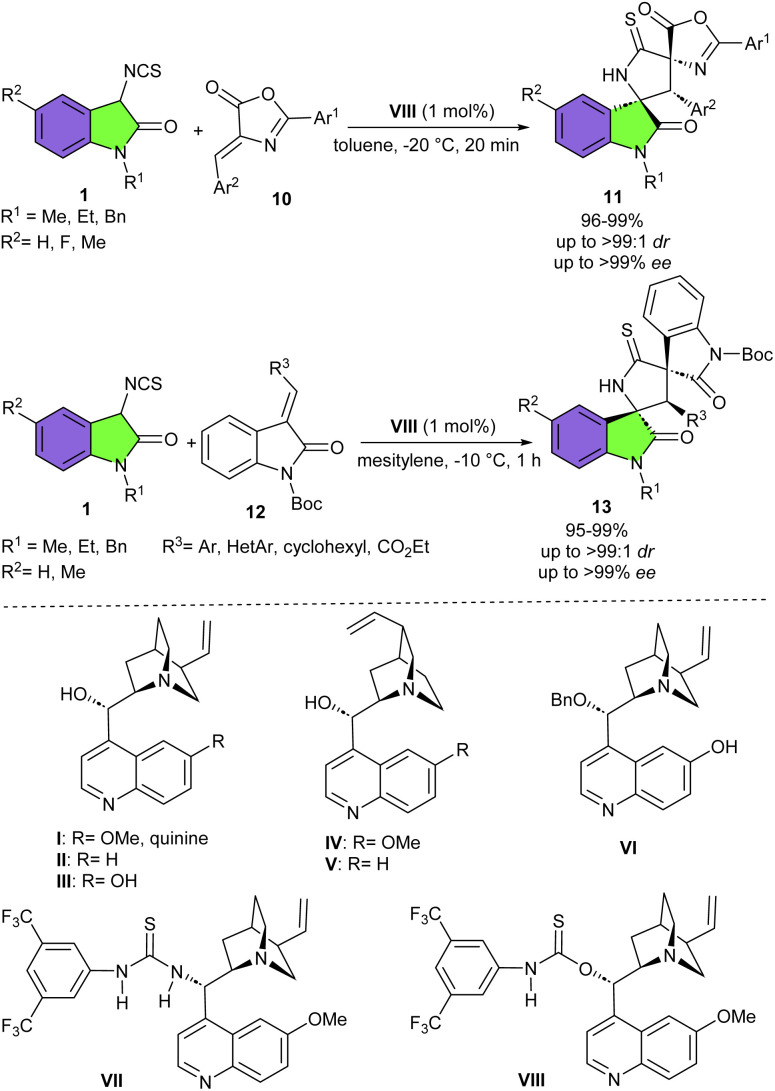
Organocatalytic reaction of 3-isothiocyanato oxindoles and (*Z*)-alkylidene azlactones.

In the same year, Yuan's group employed 1 mol% of commercially available quinine as a chiral organocatalyst in the cyclization reaction of 3-isothiocyanato oxindoles 1 and 3-methyl-4-nitro-5-alkenyl-isoxazoles 8 (Scheme 4).^14^ A domino Michael/cyclization reaction was involved, leading to diverse 3,3′-thiopyrrolidonyl spirooxindoles 9 featuring three contiguous stereogenic carbon centers in good to excellent yields (89–97%) with excellent diastereo- and enantioselectivities (up to >99 : 1 dr and up to 98% ee). Again, this research team introduced a quinine derived thiocarbamate catalyst VIII for the cyclization reaction between 3-isothiocyanato oxindole 1 and (*Z*)-alkylidene azlactones 10 (Scheme 5).^21^ Investigation of other cinchona alkaloids and their derivatives also resulted in high yields (93–98%) and diastereoselectivities (up to 98 : 2 dr) but with inverse or low enantioselectivities, indicating that enantioselectivity is strongly dependent on the structure of these catalysts. A variety of 3-isothiocyanato oxindoles with N–Me, N–Et, and N–Bn moieties reacted smoothly with (*Z*)-alkylidene azlactones, regardless of electronic or bulkiness of aromatic substitutions. The large-scale preparation of the desired product (2.11 gr, 95%, 97 : 3 dr, >99% ee), and further transformation of thiopyrrolidine oxindole into the corresponding pyrrolidinone oxindole with the high efficiency, showed the practicability of this protocol. This methodology was also successful for the synthesis of a series of biologically important spirooxindoles 13 in excellent yields and stereoselectivities (95–99%, up to >99 : 1 dr, and >99% ee) from the reaction of 3-isothiocyanato oxindoles 1 with *tert*-butyl (*E*)-3-ethylidene-2-oxoindoline-1-carboxylates 12. It should be noted that in this transformation, 3-isothiocyanato oxindole bearing N–Bn moiety showed lower enantioselectivity compared to other derivatives of 3-isothiocyanato oxindoles bearing N–Me or N–Et group, which is probably due to the steric effect of the bulky benzyl group.

**Scheme 6:**
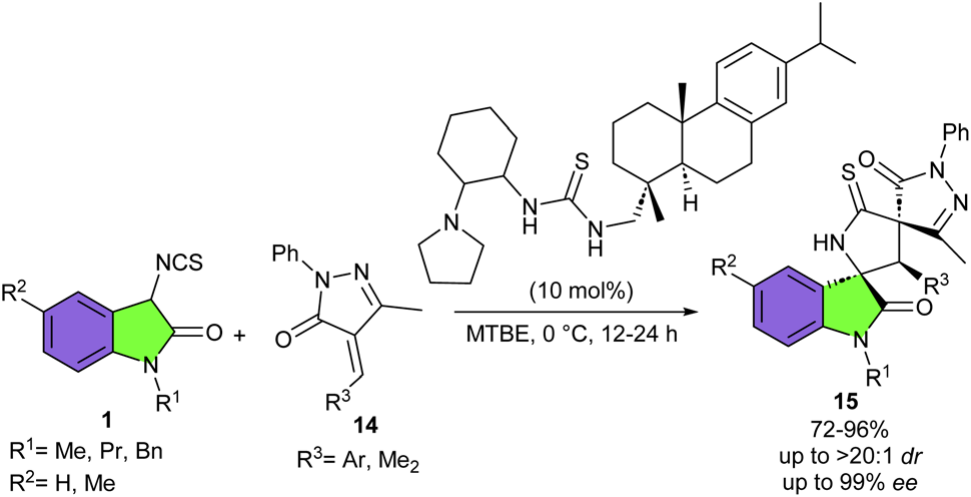
Cinchona alkaloid-derived thiourea catalyzed reaction of 3-isothiocyanato oxindoles with unsaturated pyrazolones.

**Scheme 7:**
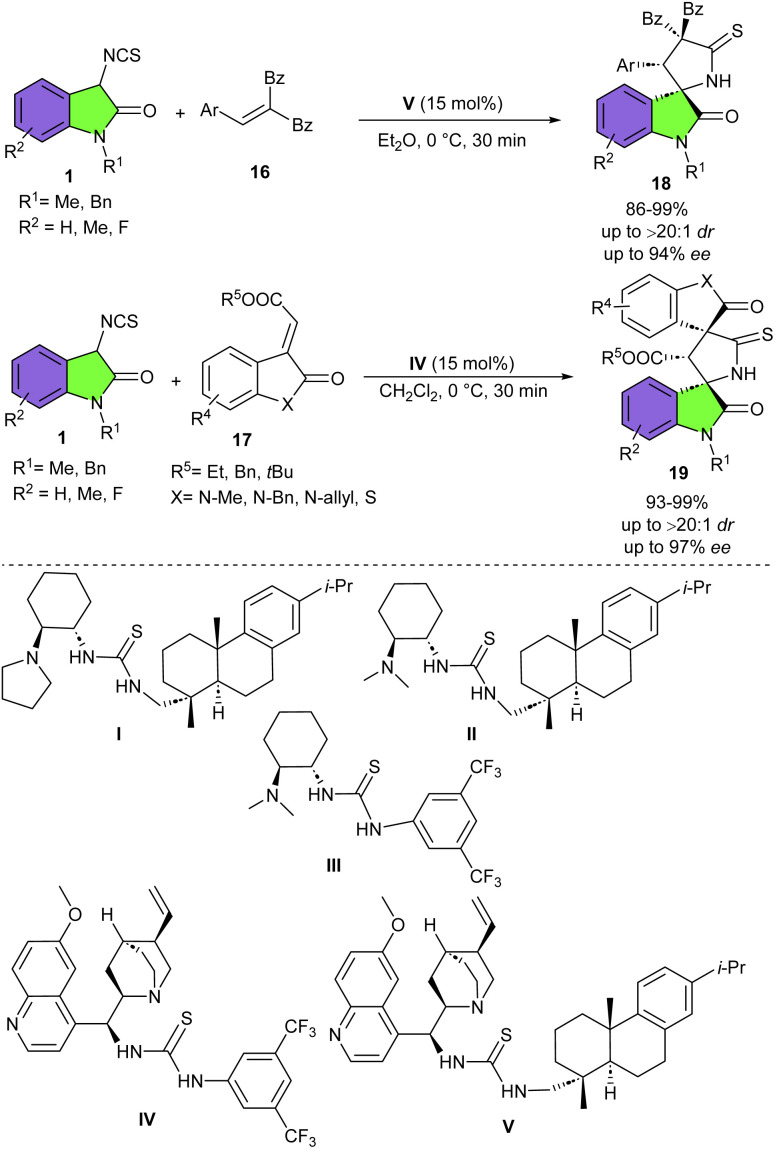
Cinchona alkaloid-derived thiourea catalyzed reaction of 3-isothiocyanato oxindoles and electron-deficient olefins.

In 2013, Wang and colleagues reported the first organocatalytic Michael/cyclization cascade reaction of 3-isothiocyanato oxindoles 1 with unsaturated pyrazolones 14 (Scheme 6).^22^ They could synthesize a new library of multicyclic spiro[oxindole/thiobutyrolactam/pyrazolone] frameworks 15 bearing three contiguous stereogenic centers with excellent diastereo- (up to >20 : 1 dr) and enantioselectivities (up to 99% ee). Screening of 3-isothiocyanato oxindoles showed that the benzyl group at the N-1 site had no effect on enantioselectivity but could reduce the diastereoselectivity more than *N*-methyl and *N*-propyl protected 3-isothiocyanato oxindoles, which could be attributed to increased steric hindrance. For unsaturated pyrazolones, electron-donating and electron-withdrawing groups at different positions of the phenyl ring afforded the corresponding spirooxindoles in high yields, good diastereoselectivities, and excellent enantioselectivities. Moreover, a gram-scale synthesis of spirooxindole was also performed in 90% yield, 14 : 1 dr and 90% ee. In another work by Wang *et al.*, a series of electron-deficient olefins were used as coupling partner for 3-isothiocyanato oxindoles to make 3,2′-pyrrolidinyl spirooxindole products in up to 99% yield with >20 : 1 dr, and 96% ee (Scheme 7).^23^ Among organocatalysts I–V, catalysts I–IV resulted in lower enantioselectivities, while cinchona alkaloid-derived thiourea bifunctional organocatalyst V with bulky dehydroabietic amine moieties was found to be better choice for the asymmetric Michael addition/cyclization reaction between 3-isothiocyanato oxindoles 1 and electron-deficient olefin 16. While organocatalyst IV was effective for the preparation of enantioenriched 3,2′-pyrrolidinyl bi-spirooxindoles 19, when electron-deficient olefins 17 were used as reactants in the reaction with 3-isothiocyanato oxindoles 1. The hypothesis was that the smaller organocatalyst IV could perform more effectively in the crowded transition state containing bulkier electron-deficient olefins. Electron-donating and electron-withdrawing groups in 3-isothiocyanato oxindoles all were compatible and led to the generation of the cycloadducts with good results.

**Scheme 8:**
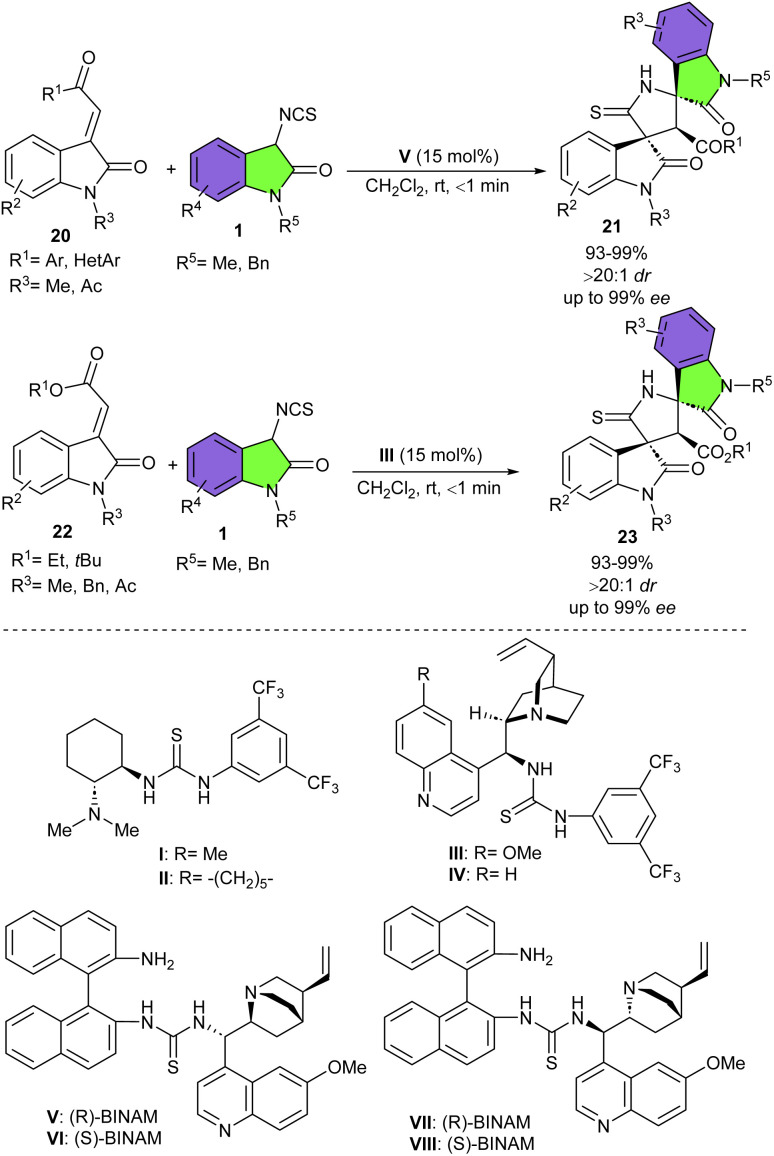
Bi- or multifunctional cinchona alkaloids catalyzed reaction of 3-isothiocyanato oxindoles and methyleneindolinones.

Another group developed an elegant organocatalytic strategy for the synthesis of stereodivergent bispirooxindole skeletons 21, 23 from 3-isothiocyanato oxindoles (Scheme 8).^24^ They treated 3-isothiocyanato oxindoles 1 with methyleneindolinones 20, 22 in the presence of different organocatalysts, where all of the catalysts led to high to excellent yields of products (89–98%) in less than one minute. While in the point of view of stereoselectivity, bifunctional catalyst III and trifunctional catalyst V displayed more acceptable enantioselectivities. It was found that two chiral elements of organocatalyst V play a key role in controlling the stereoselectivity of Michael-cyclization reaction between 3-isothiocyanato oxindoles with methyleneindolinones. However bifunctional cinchona alkaloid III showed good catalytic action for the synthesis of bispirooxindoles with ester moieties. It should be noted that the opposite enantiomer can be accessed by reconfiguring the catalyst. Generally, negligible impact was observed on the reactivities, and stereoselectivities, regardless of the electronic nature, bulkiness, or positions of the substituents at the aryl ring, or even N1-substituents in both methyleneindolinones and 3-isothiocyanato oxindoles as substrates. When the experiment was performed on a preparative-scale, 1.14 gr of the desired product was obtained in 98% yield, >20 : 1 dr, and 98% ee. Further transformations of γ-thiolactam moiety of bispirooxindole into γ-lactam and methylated thiolactam were also established in this work.

**Scheme 9:**
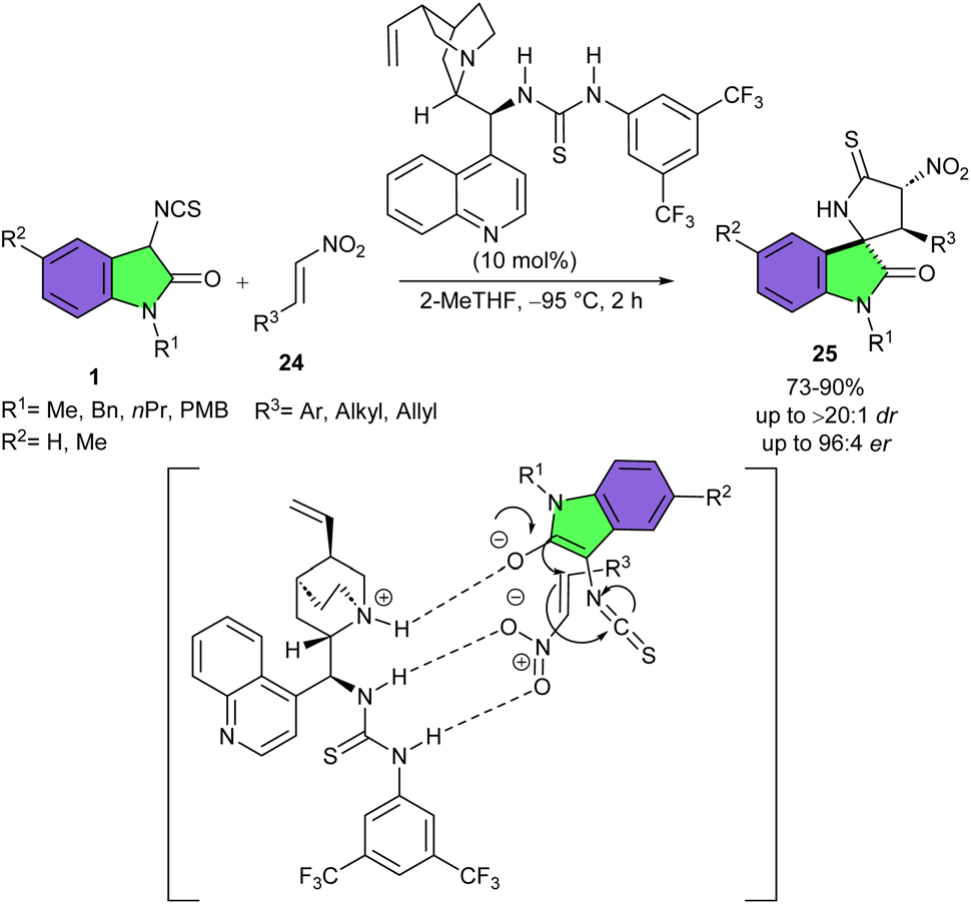
Quinine-catalyzed reaction of 3-isothiocyanato oxindoles and imines.

In very mild reaction conditions (−95 °C), a series of highly functionalized 3,2′-pyrrolidinyl-substituted spirooxindoles 25 could be synthesized from reaction of 3-isothiocyanato oxindoles 1 with nitro olefins 24 using a cinchonidine-derived bifunctional catalyst (Scheme 9).^25^ High yields of products with moderate to good diastereo- and enantioselectivities (up to >20 : 1 dr and up to 96 : 4 er) were observed under this catalytic asymmetric Michael addition/cyclization cascade reaction. Several N–H 3-isothiocyanato oxindoles bearing different substituents on the aryl ring as well as N–Me derivative of 3-isothiocyanato oxindole were well tolerated in this reaction. Generally, aryl and heteroaryl substituents at the C=C moiety of β-monosubstituted nitro olefins drastically showcased better reactivities and stereoselectivities compared to alkyl substituents, especially the bulky iso-butyl group. Besides, the obtained products showed instability during prolonged storage, thus the authors could not use perform further synthetic transformations. According to the transition state, the thiourea moiety of the organocatalyst activated the nitro olefin by forming two hydrogen bonds. Then, the Brønsted basic tertiary amine of the organocatalyst nucleophilically attacked 3-isothiocyanatooxindole, leading to the selective addition of 3-isothiocyanatooxindole to the Re face of nitro olefin, followed by cyclization step to form spirooxindole 3.

**Scheme 10:**
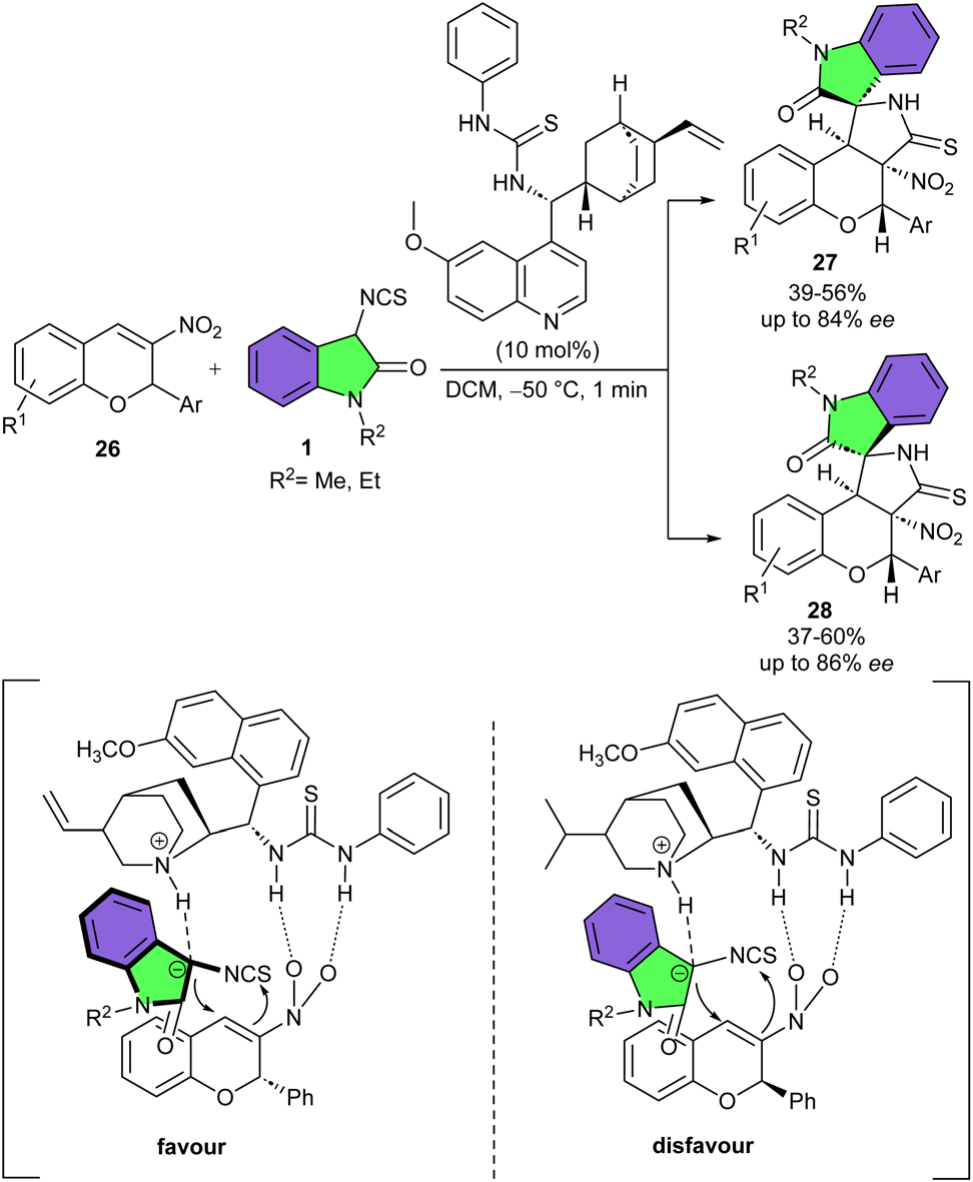
Thiourea-catalyzed reaction of 3-isothiocyanato oxindoles and nitro olefins.

An asymmetric domino reaction was reported between 3-nitro-2*H*-chromenes 26 and 3-isothiocyanato oxindoles 1 (Scheme 10).^26^ A mixture of two enantiomers of tetracyclic spiro[chromeno[3,4-*c*] pyrrole-1,3′-indoline]s were obtained using a bifunctional thiourea organocatalyst. Good efficiency and enantioselectivity were observed whether electron-withdrawing or electron-donating substituents were introduced on the aryl ring 3-nitro-2*H*-chromenes. However, the *ortho*-substituted 3-nitro-2*H*-chromene resulted in slightly inferior enantioselectivity, presumably due to the steric effect. The substrate scope with respect to 3-isothiocyanato oxindoles was limited to the introduction of methyl and ethyl protecting groups on the nitrogen atom. Both derivatives showcased good reactivity and stereoselectivity. Notably, the spirocyclic product 27 can also be prepared when the adduct 28 is treated in the presence of DABCO in ethanol at 15 °C after a prolonged period.

Based on the X-ray analysis of two enantiopure derivatives, a plausible transition state model was proposed, involving the activation of both substrates by the catalyst. The activated Michael acceptor was oriented by the hydrogen bonding interactions with the bifunctional thiourea. Meantime, the tertiary amine of the catalyst provided suitable basicity to enhance the nucleophilicity of 3-isothiocyanato oxindole. The well-defined orientation allowed the Re attack on the activated 3-nitro-2*H*-chromene, which favored the formation of the C2*S* stereocenter. Next intramolecular cyclization through the attack the isothiocyanato group produced the major C3*S* product.

**Scheme 11:**
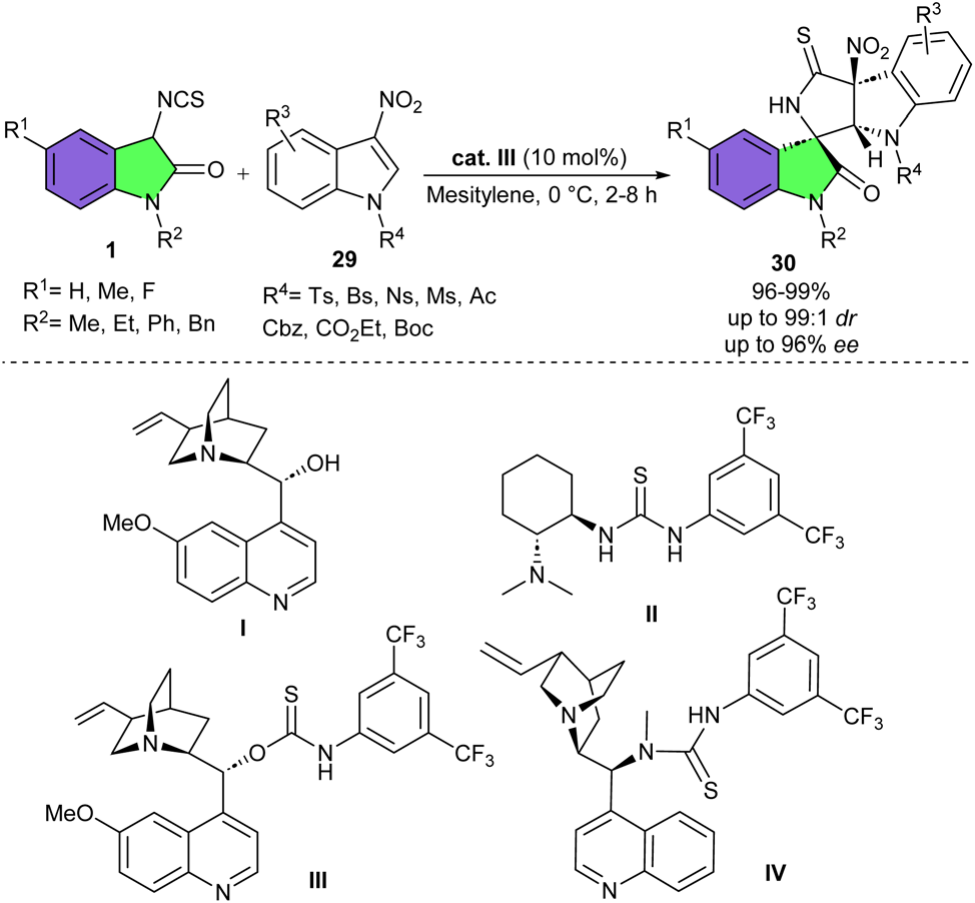
Amino-thiocarbamate-catalyzed reaction of 3-isothiocyanato oxindoles and 3-nitroindoles.

In 2015, Yuan's research group conducted the reaction of 3-isothiocyanato oxindoles 1 with 3-nitroindoles 29 in the presence of different amino-thiocarbamate catalysts (Scheme 11).^27^ All four catalysts led to the excellent yields (97–99%), but with varied stereoselectivities. The best diastereo- and enantioselectivity belonged to organocatalyst III. Electron-withdrawing and electron-donating substituents at the C5, C4-, C6-position of 3-nitroindoles showed very high reactivities, providing the corresponding products in excellent results. 3-Isothiocyanato oxindoles bearing different N-protecting groups gave good diastereoselectivities and moderate enantioselectivities, indicating the crucial importance of the steric hindrance of the N1-position of 3-isothiocyanato oxindoles for the stereocontrol ability of the chiral catalyst. For 3-isothiocyanato oxindole with electron-withdrawing or electron-donating group, no notable effect was observed in the reactivity and selectivity of the reaction. To show the synthetic application of this asymmetric transformation, two large-scale experiments were performed using different amounts of the oegano catalyst III. The reaction proceeded with 10 mol% of III furnished 1.03 gr of the spirocyclic product 99% yield, >99 : 1 dr, and 96% ee, whereas 1 mol% of III produced 0.5 gr of the product in 96% yield, 97 : 3 dr, 94% ee.

**Scheme 12:**
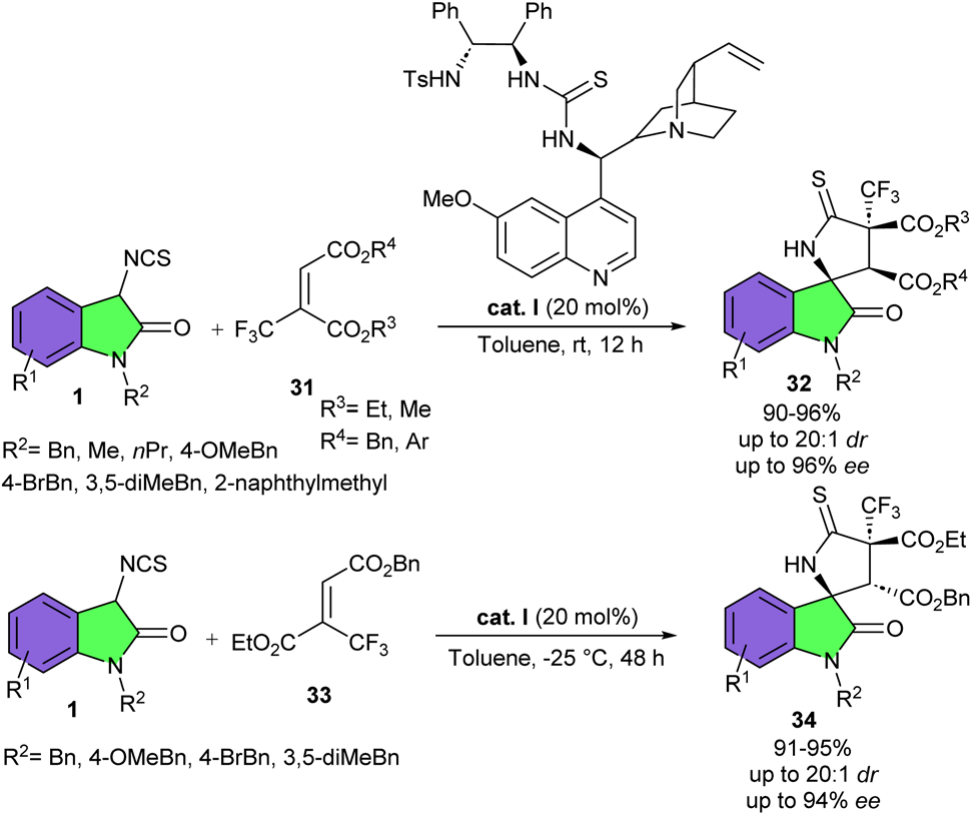
Cinchona alkaloid derived multifunctional amine catalyzed reaction of 3-isothiocyanato oxindoles and trifluoromethylated 2-butenedioic acid diesters.

**Scheme 13:**
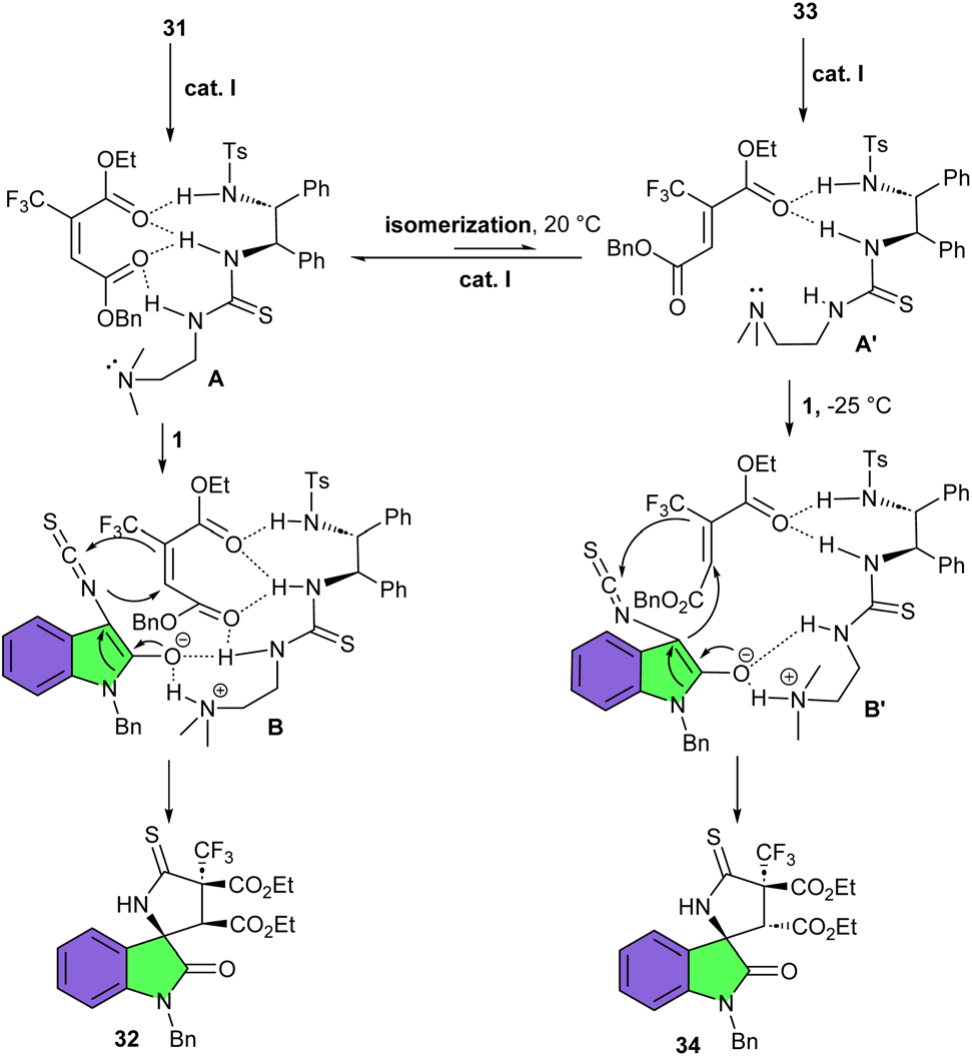
Possible mechanism for cinchona alkaloid derived multifunctional amine catalyzed reaction of 3-isothiocyanato oxindoles and trifluoromethylated 2-butenedioic acid diesters.

Trifluoromethylated 2-butenedioic acid diesters 31 can be used a coupling partners for (3 + 2)-cycloaddition reaction with 3-isothiocyanato oxindoles 1 for the preparation of spirooxindoles 32 bearing a CF_3_-containing quaternary carbon stereocenter (Scheme 12).^28^ Among various multifunctional organocatalysts derived from cinchona alkaloids, cinchona alkaloid derived organocatalyst I was found to be the best catalyst for the reaction, producing spirocyclic products in high yields (91–96%) with excellent diastereo- and enantioselectivities (up to 20 : 1 dr, and up to 96% ee). The reaction did not affect by electronic and steric properties of functional groups at the aryl ring of 3-isothiocyanato oxindoles. Although the presence of the sterically bulky protecting group such as a benzyl at N1-position, could slightly enhance the enantioselectivity. The regulation of the temperature was found to be very important, as two epimeric isomers could be obtained with the same organocatalyst at different temperatures. This method is an enantiodivergent approach for the synthesis of spirooxindoles. As shown in the mechanism, catalyst I formed two hydrogen bonds with both ester groups in 31 and synchronously abstracted an acidic proton from 3-isothiocyanato oxindole 1 to render intermediate B. Then, B underwent an intermolecular Michael addition/cyclization to form product 32. For ester 33, organocatalyst I interacted with only one ester group, which could partially isomerize to 31 at room temperature (20 °C) to furnish 32 as the major product. While, at −25 °C, product 34, an epimer of 32, was exclusively formed *via* intermediate B′ (Scheme 13).

**Scheme 14:**
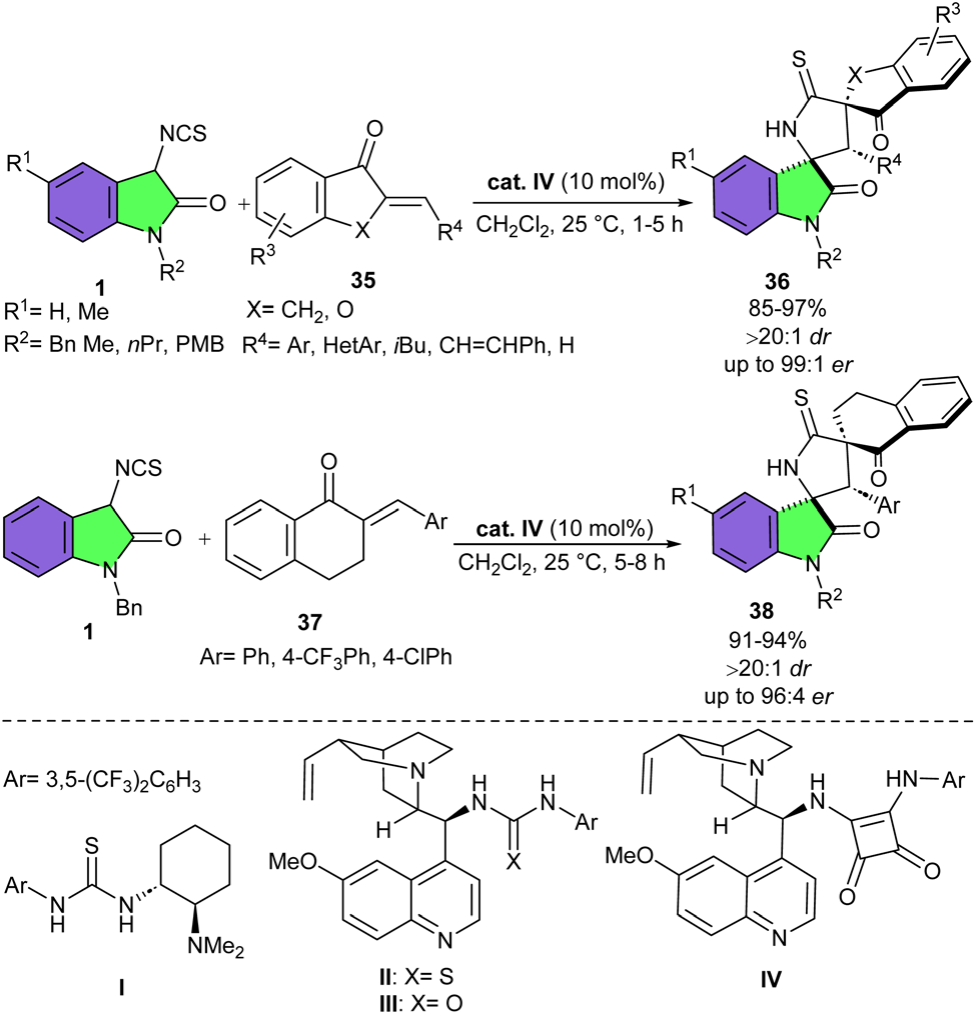
Organocatalytic cascade Michael addition/cyclization reaction between 3-isothiocyanato oxindoles and exocyclic α,β-unsaturated ketones.

In 2016, Mukherjee and Kayal developed an organocatalytic sequential Michael addition/cyclization reaction between 3-isothiocyanato oxindoles 1 and (*E*)-β-benzylidene-α-indanones 35 (Scheme 14).^29^ Evaluation of four organocatalyst candidates showed that Takemoto catalyst I led to the desired product with good diastereoselectivity but moderate enantioselectivity. Quinine-derived thiourea II was less diastereoselective but more enantioselective. Urea-derived catalyst III had similar level of selectivities, whereas the quinine-derived tertiary amino-squaramide IV displayed better results, furnishing 3,2′-pyrrolidinyl bispirooxindoles 36 in high yields (86–97%) with complete diastereoselectivity and excellent enantioselectivity (up to 99 : 1 er). A survey of solvents revealed CH_2_Cl_2_ is the best, producing spirocyclic product 36 as a single diastereomer with 98 : 2 er. Various β-arylidene-α-indanones, with different steric and electronic characters, and β-alkylidene-α-indanones were compatible, although the enantioselectivity was found to be slightly lower in β-alkylidene-α-indanones. 3-Isothiocyanato oxindoles featuring alkyl, benzyl, or PMB at the N1-position, all showed high reactivities and selectivities. The synthetic potential of this method was then investigated on a larger scale. The target spirocycle was obtained in 495 mgr, and 98% yield, with the same level of stereoselectivity (>20 : 1 dr, 98 : 2 er). In addition, this enantioselective Michael addition/cyclization reaction was also tested for three derivatives of β-arylidene-α-tetralones 37 in the reaction with 3-isothiocyanato oxindoles 1. The corresponding spirocycles 38 were obtained in 91–94% yield, >20 : 1 dr, and up to 96 : 4 er.

**Scheme 15:**
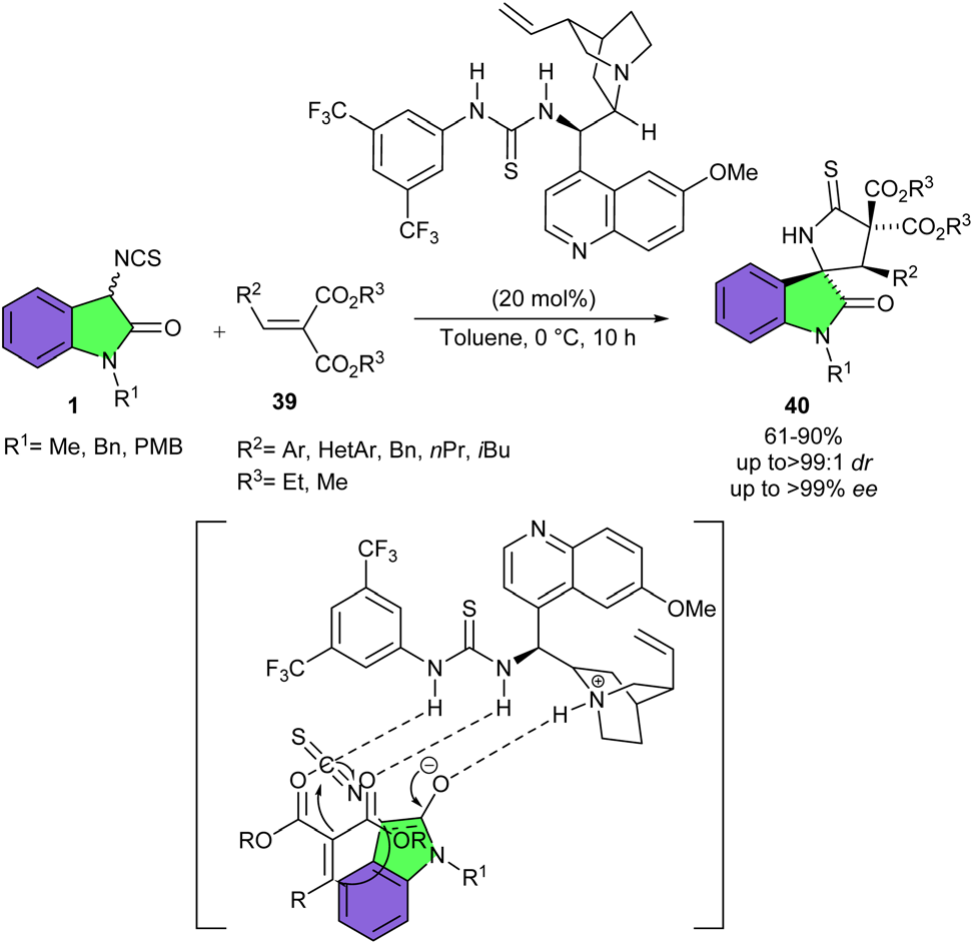
Quinine derived tertiary amino-thiourea catalyzed of 3-isothiocyanato oxindoles and arylidene malonates.

Chowdhury *et al.* reported the first example of organocatalyzed cascade Michael addition/cyclization reaction of 3-isothiocyanato oxindoles 1 with arylidene malonates 39 as a relatively less reactive π-electrophile (Scheme 15).^30^ The preparation of highly functionalized 3,2′-pyrrolidinyl spirooxindole structures 40 was achieved in high yields and with excellent diastereo- (up to 99 : 1 dr) and enantioselectivities (up to >99% ee) using a quinine derived tertiary amino-thiourea based bifunctional catalyst. The pseudo-enantiomeric quinidine derived catalyst could also provide both the enantiomers of the desired product. The nature of the ester group in the arylidene malonates showed significant impact on the yield and enantioselectivity. Where the methyl esters of arylidene malonates led to higher yield and enantioselectivity compared to the ethyl esters. For 3-isothiocyanato oxindoles, the substituent group at nitrogen of had negligible influence on the reaction. The survey of alkylidene malonates as acceptors was also successful and gave the corresponding products in good yield and diastereoselectivity, but with slightly inferior enantioselectivity compared to arylidene malonate. The authors proposed a plausible transition state for this reaction, involving the activation of the electrophile (arylidene malonate) by the thiourea moiety of the catalyst. Simultaneously, the Brønsted basic tertiary amine of the catalyst deprotonated the α-H next to the isothiocyanato group of 3-isothiocyanatooxindole. Thus, the generated enolate underwent Michael addition from its Si face to the Si face of the arylidene malonate, followed by intramolecular cyclization to yield product 40.

**Scheme 16:**
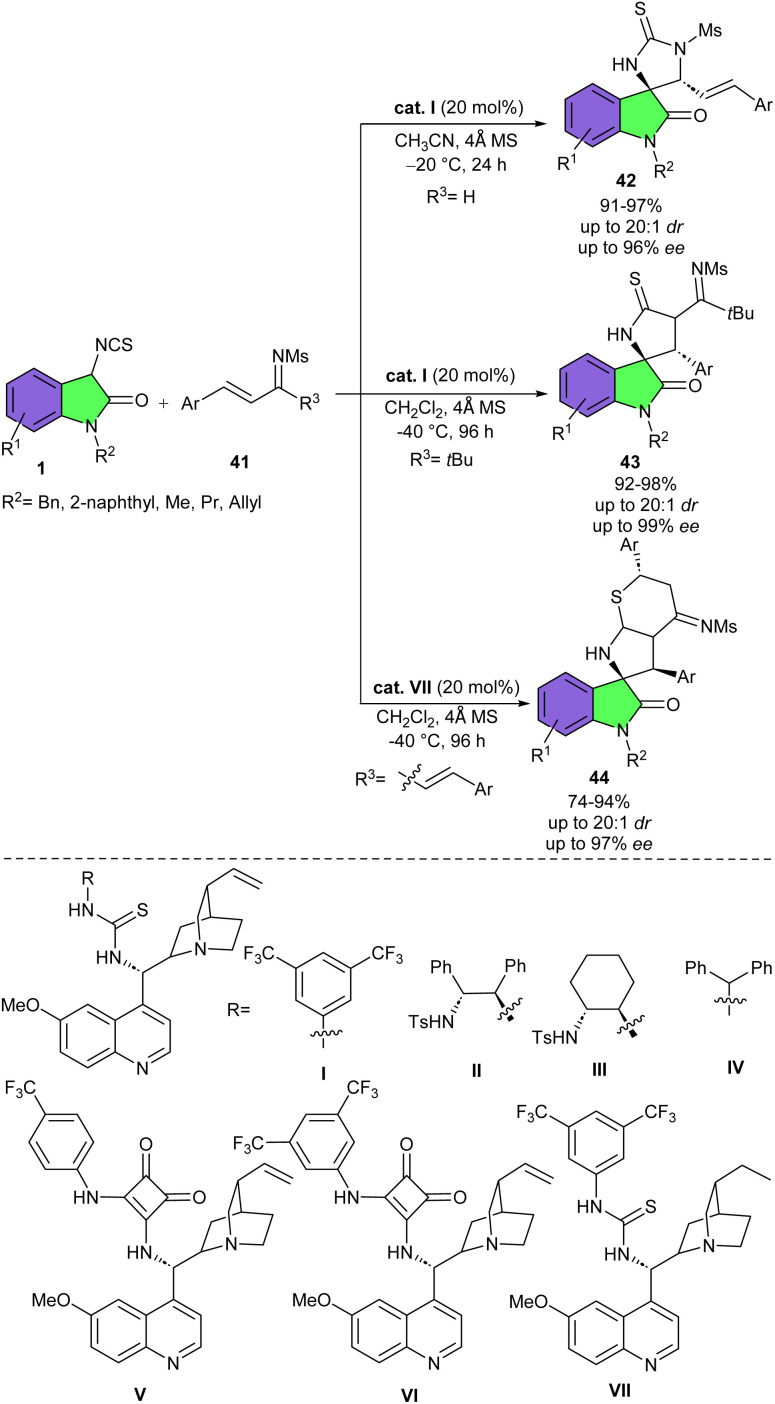
Cinchona alkaloid-derived organocatalyst catalyzed reaction of 3-isothiocyanato oxindoles and α,β-unsaturated methanesulfonamides.

**Scheme 17:**
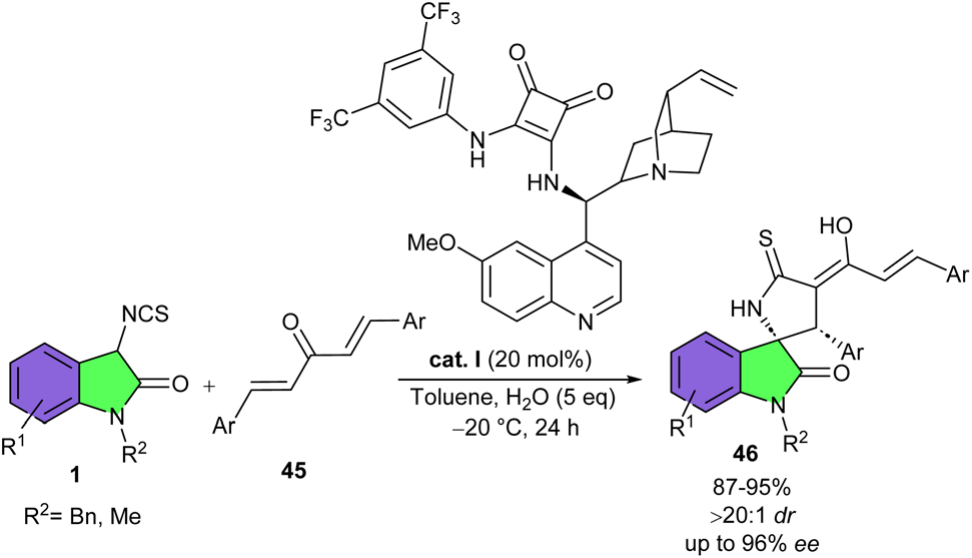
Cinchona-alkaloid-derived organocatalyst catalyzed reaction of 3-isothiocyanato oxindoles and dibenzylidene ketones.

**Scheme 18:**
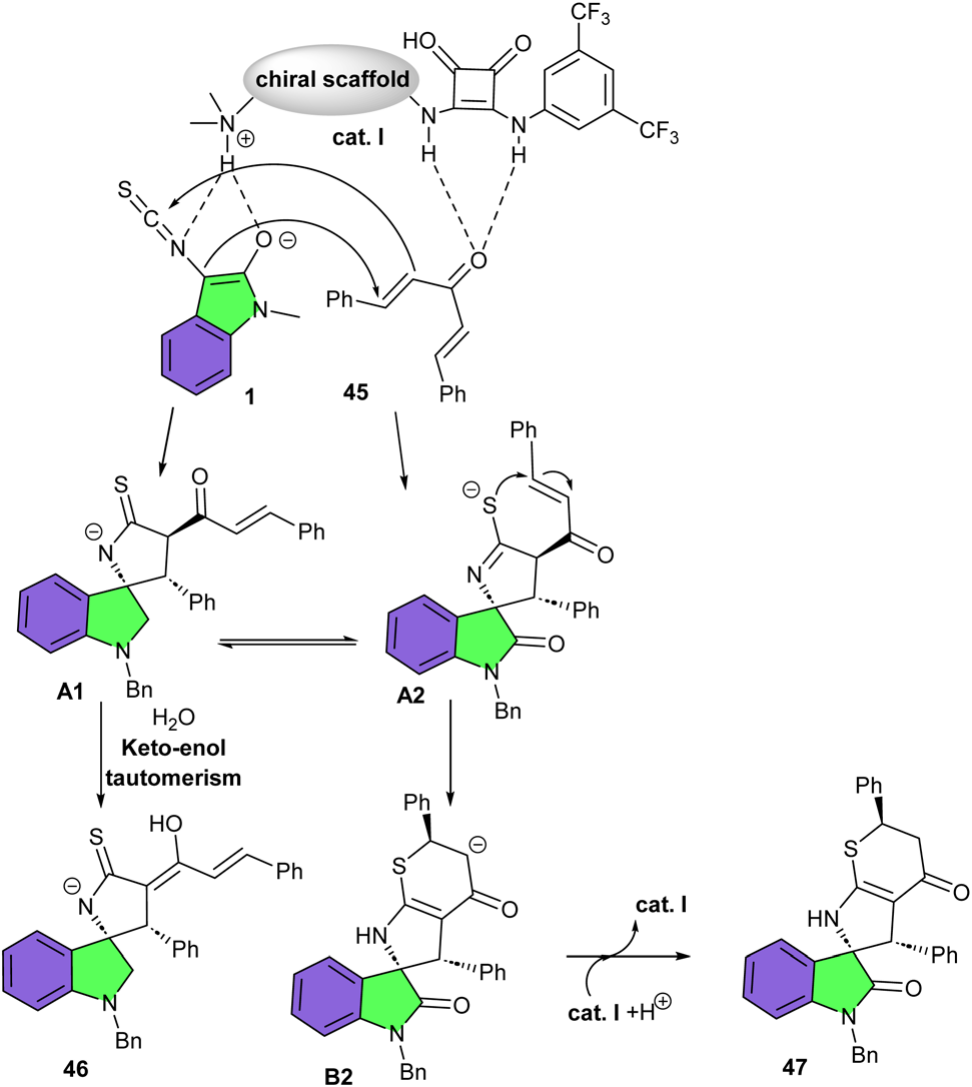
Possible mechanism for organocatalysis reaction of 3-isothiocyanato oxindoles and dibenzylidene ketones.

In 2016, Shi's research lab reported asymmetric regioselective (3 + 2) or (3 + 2)/(4 + 2) cascade cycloaddition reaction of 3-isothiocyanato oxindoles with C=C and C=N bonds of α,β-unsaturated aldimines/ketoimines and imines (Scheme 16).^31^ Various cinchona alkaloid-derived organocatalysts were tested for the synthesis of diversified S-containing heterocyclic spirooxindole derivatives 42. All organocatalysts resulted in high yields (90–93%) but moderate stereoselectivities. Among them, catalyst I represented better catalytic activity in the (3 + 2) cycloaddition reactions of 3-isothiocyanato oxindoles 1 with α,β-unsaturated aldimines (R^3^ = H) or ketoimine (R^3^ = *t*Bu). In such transformations, two reactive sites of 3-isothiocyanato oxindoles participated in regio- and stereocontrolled (3 + 2)-cycloaddition reaction of with C=C and C=N bonds of α,β-unsaturated aldimines and ketoimines, affording spirocycles 42 and 43, respectively. While catalyst VII was found to be suitable for the (3 + 2)/(4 + 2) cyclization reaction of 3-isothiocyanato oxindoles 1 with α,β-unsaturated methanesulfonamides (R^3^ = CH_2_=CHAr). When three reactive sites of 3-isothiocyanato oxindoles reacted with electron-deficient C=C bonds of α,β-unsaturated imines, regio- and stereocontrolled (3 + 2)/(4 + 2) cascade cycloaddition took place, yielding spirooxindoles 44. It was found that the C=S and C=N bonds in such spirooxindoles can be easily converted to the C=O bond under mild conditions and after oxidation, which enriches the derivatization method of spirooxindoles. Again, Shi and co-workers accomplished regioselective (3 + 2)-cycloaddition reaction between 3-isothiocyanato oxindoles 1 and dibenzylidene ketones 45 (Scheme 17).^32^ For this purpose, they used a new cinchona-alkaloid-derived organocatalyst I to assembly spirooxindole enols 46 in high yields (87–95%) with high diastereo- and enantioselectivities (up to >20 : 1 dr, and 96% ee). Decreasing the reaction temperature from rt to −20 °C improved the enantioselectivity of the spirocyclic product. Also, adding water could suppress the formation of a S-containing heterocyclic spirooxindole, which could be formed *via* a (3 + 2)/(4 + 2)- cycloaddition reaction of three active sites of 3-isothiocyanato oxindole 1 with dibenzylidene ketone 45. Thus, spirooxindole enol was formed as the only product. Based on the observations, a plausible pathway for the formation of product 3 and 4 was proposed, which begins with the activation of 1 and 45 by hydrogen bond donor site and the amine base site of organocatalyst I, respectively. Subsequent Michael addition/cyclization reaction led to intermediate A1 or A2. Intermediate A1 was protonated in the presence of H_2_O and the keto–enol tautomerism gave the enol product 46. On the other hand, in the absence of H_2_O, intermediate A2 moved through further Michael addition/cyclization and protonation to yield product 47 (Scheme 18).

**Scheme 19:**
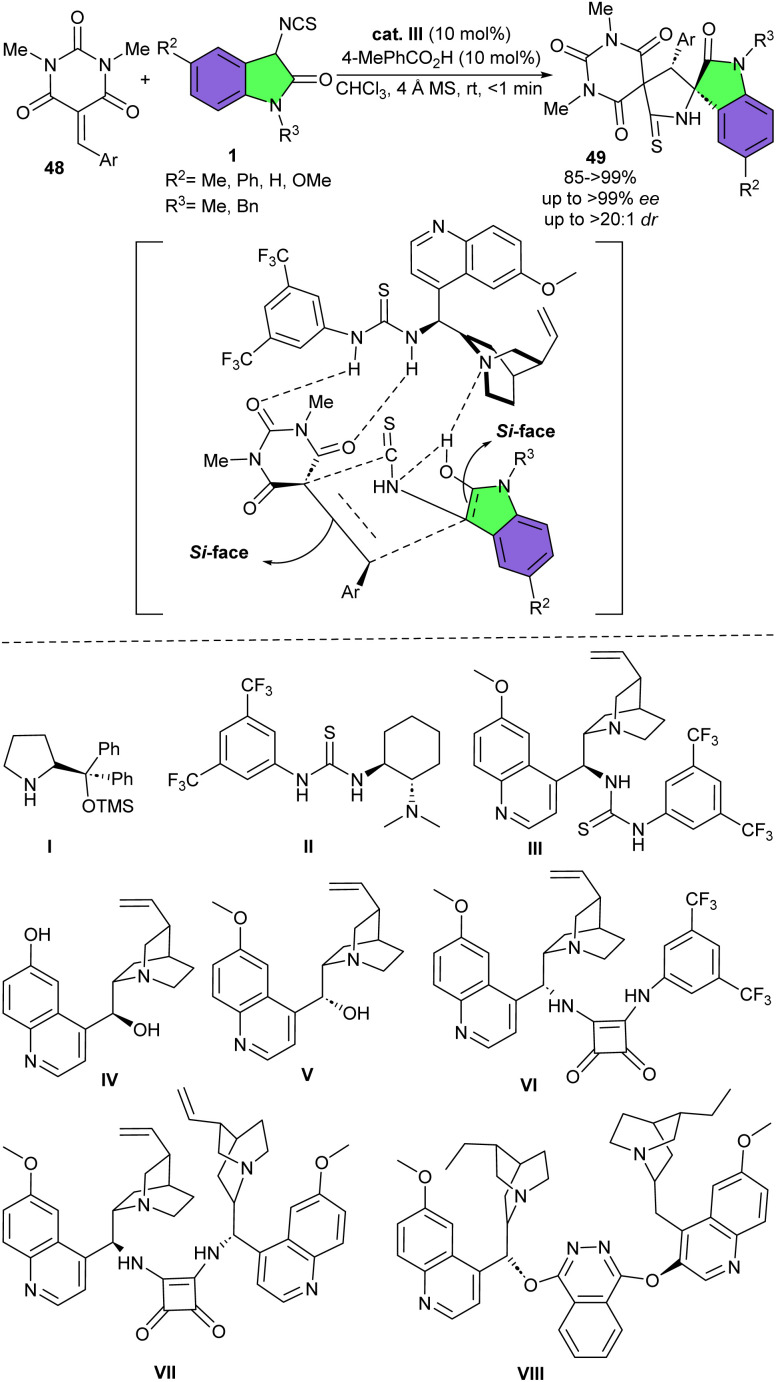
Organocatalysis reaction of 3-isothiocyanato oxindoles and barbiturate-based olefins.

A wide range of organocatalysts were studied for the stereoselective (3 + 2)-cycloaddition of 3-isothiocyanato oxindoles 1 with barbiturate-based olefins 48 (Scheme 19).^33^ In all cases, the cycloaddition led to the spirocyclic product 49 in almost same excellent diastereoselectivity, but different yield and enantioselectivity. For example, catalyst IV afforded a racemate product in moderate yield. The use of V increased the yield and enantioselectivity. In the presence of catalysts VI and VIII, the opposite enantiomer was formed. With respect to organocatalysts I, II and VII, the cycloaddition furnished spirocycles in good to excellent yields with moderate to high enantioselectivities. Using III, the cycloaddition afforded the product in >99% chemical yield with 80% ee, which showed best result. In addition, the authors noted that the addition of 4 Å MS could enhance enantioselectivity by diluting the reaction medium. 3-Isothiocyanato oxindoles bearing electron-donating groups on the phenyl ring generated the desired product in significantly higher enantioselectivity, than those with electron-withdrawing substituents. Also, sterically bulky benzyl group in N1 of 3-isothiocyanato oxindole had good impact on the cycloaddition, by increasing the enantioselectivity in comparison with 3-isothiocyanato oxindole with the less steric methyl group.

**Scheme 20:**
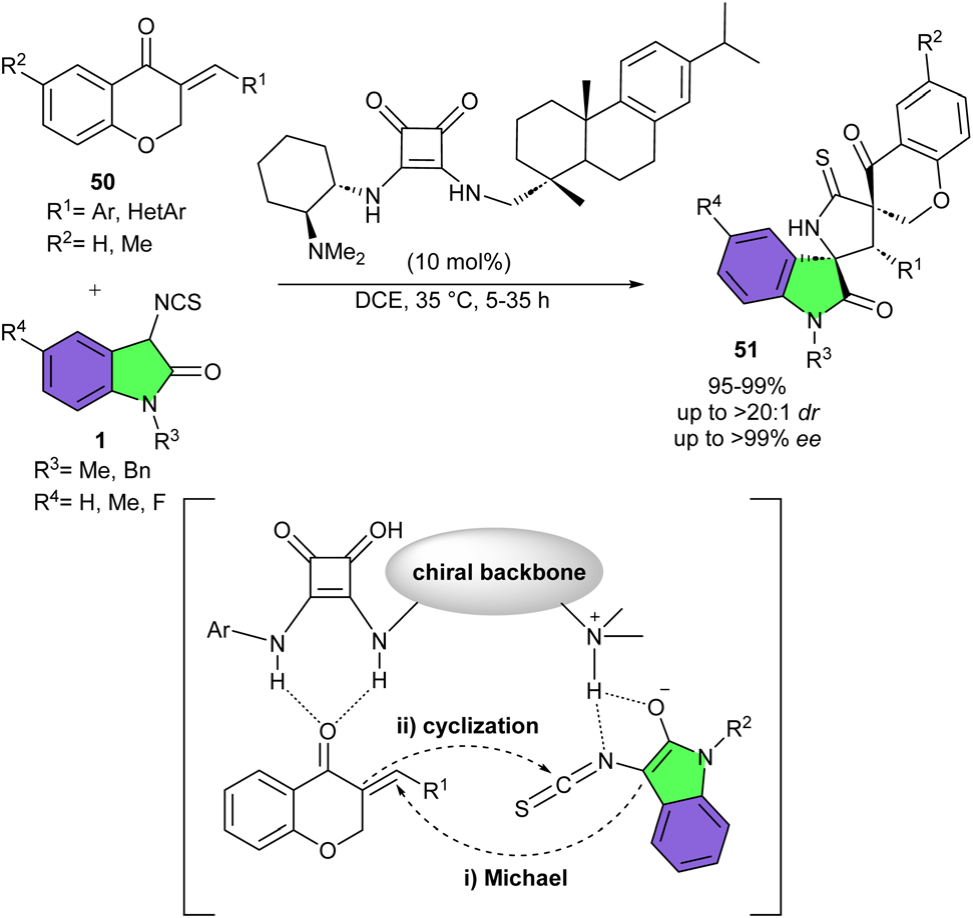
Organocatalysis reaction of 3-isothiocyanato oxindoles and 4-chromanones.

Asymmetric Michael/cyclization cascade reaction of 3-isothiocyanato oxindoles 1 with 4-chromanones 50 can lead to the synthesis of a range of spiro[oxindole/thiobutyrolactam/chromanone] derivatives 51 (Scheme 20).^34^ Neutral 3-isothiocyanato oxindole with Me group on the nitrogen, or bearing Me or F groups on the benzene ring of oxindole reacted smoothly with a wide range of electron-rich and electron-poor 4-chromanone derivatives affording the corresponding spirocyclic products in high to excellent yields with excellent stereoselectivities (97–99%, >20 : 1 dr and >99% ee). It was found that the position and electronic properties of each substituent on the benzene ring of 4-chromanone have a negligible effect on enantioselectivity. Moreover, 3-isothiocyanato oxindole featuring Bn group on the nitrogen also gave the desired product in high yield and diastereoselectivity, but moderate enantioselectivity (95%, >20 : 1 dr and 60% ee). Featuring A unprecedented rosin-based squaramide organocatalyst was introduced for constructing spirocyclic compounds possessing three contiguous stereocenters and two spiro-quaternary. Hydrogen bonding interactions of the organocatalysts with both substrates could approach them for subsequent Michael addition and cyclization reactions. The gram-scale preparation of the product was also performed in high yield (1.15 gr, 91%) without any decrease in stereoselectivity (>20 : 1 dr, 99% ee).

**Scheme 21:**
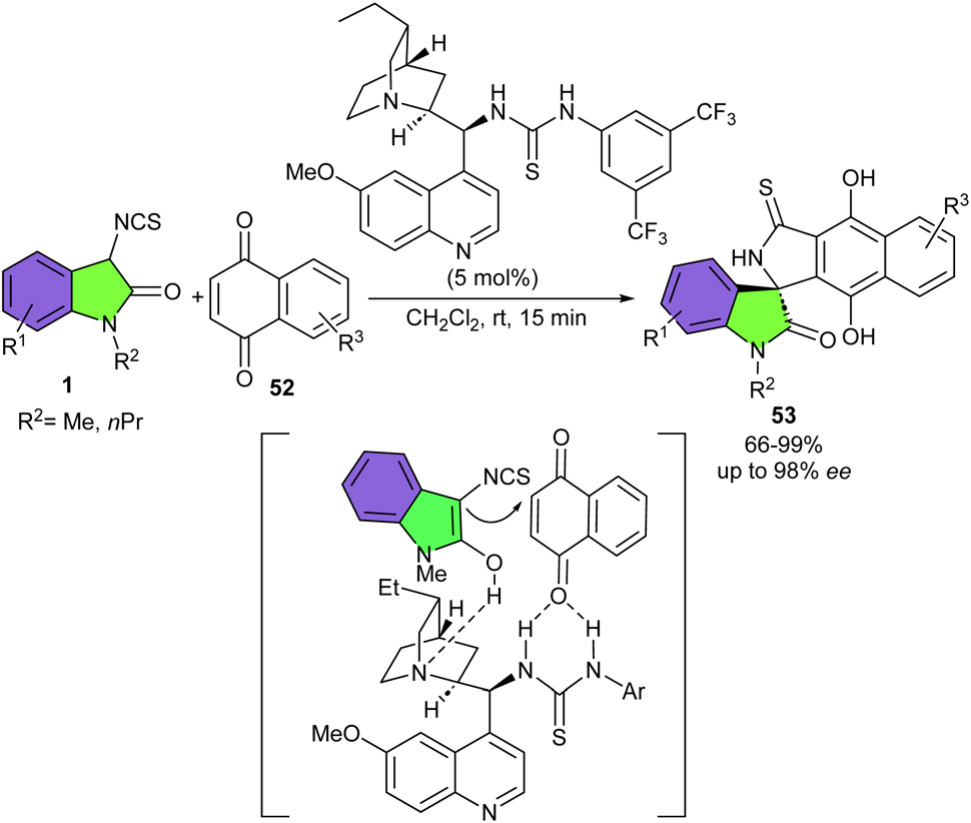
Organocatalysis reaction of 3-isothiocyanato oxindoles and quinones.

Quinones 52 could be used as the electrophiles in Michael addition/cyclization cascade reaction with 3-isothiocyanato oxindoles 1 (Scheme 21).^35^ A chiral cinchona alkaloid derived catalyst promoted this reaction effectively to provide spirocyclic products 53 in high to excellent yields (80–99%) with high enantioselectivities (up to 97% ee). The reaction of neutral 3-isothiocyanato oxindoles or with Me, OMe or di-Me substituents gave rise the corresponding products in excellent yield and enantioselectivity, while the oxindole possessing F substituent led to slightly lower enantiomeric excess. Replacing the protecting group of oxindole from Me to *n*Pr or Bn provided the products in excellent yields, albeit lower enantioselectivities. In addition, 1,4-naphthoquinone and anthracene-1,4-dione as coupling partners provided the spirocycles in good to excellent yields with good enantioselectivities. While quinone derivative delivered the product in high yield (90%), with moderate enantioselectivity (66% ee). To enhance the enantioselectivity for two products, the authors applied a chiral phosphoric acid as the catalyst. The corresponding spirocycles were obtained with higher enantioselectivities but lower yields. Finally, the gram-scale reaction of this method was studied, showing the desired product in 1.377 gr, 95% yield with 93% ee. In the plausible transition state model, the bifunctional organocatalyst activated both 3-isothiocyanato oxindole and quinone *via* multiple hydrogen bonding interactions to allow double arylation processes. Meanwhile, the chiral backbone of the organocatalyst provided an ideal environment to introduce the chirality.

**Scheme 22:**
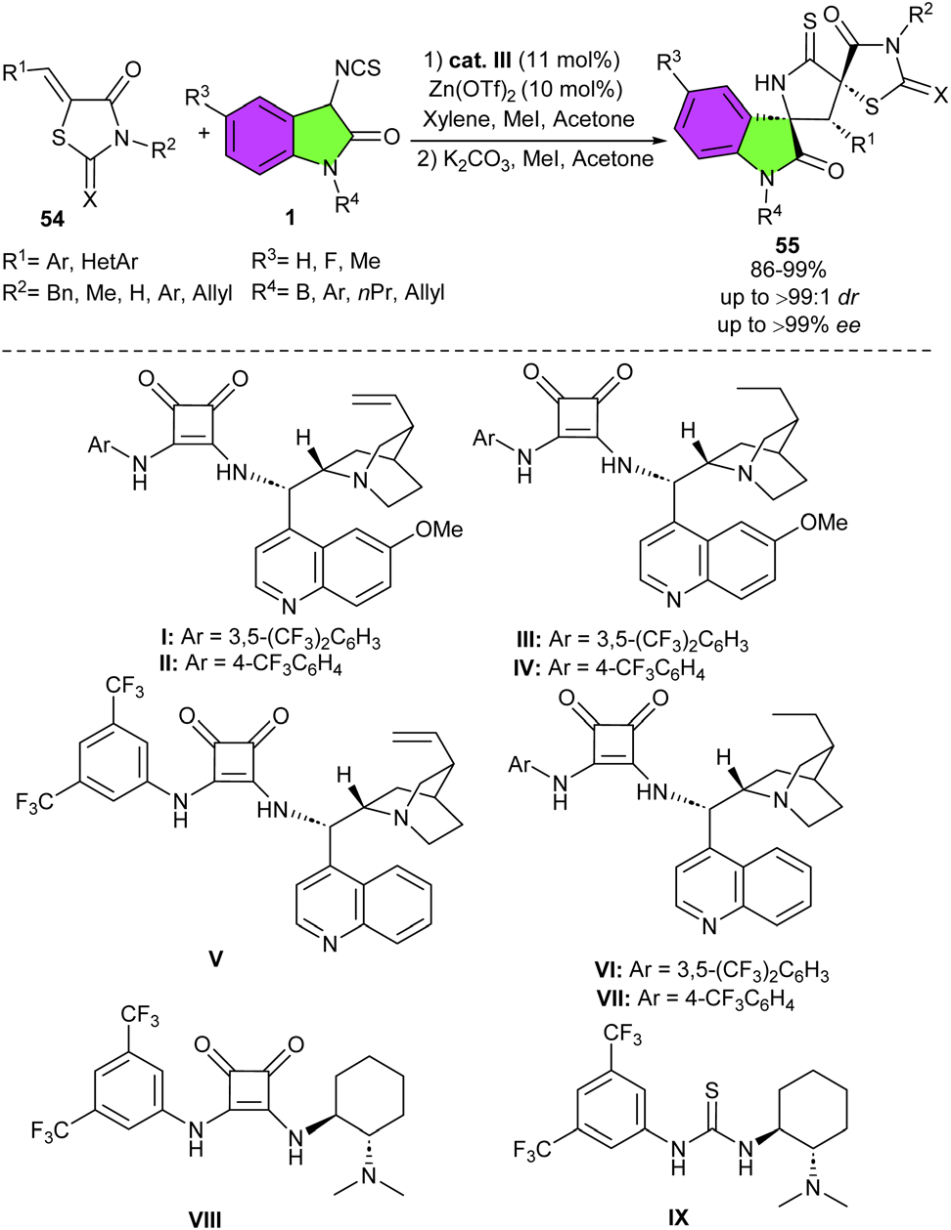
Organocatalysis reaction of 3-isothiocyanato oxindoles and unsaturated thiazolidinones.

**Scheme 23:**
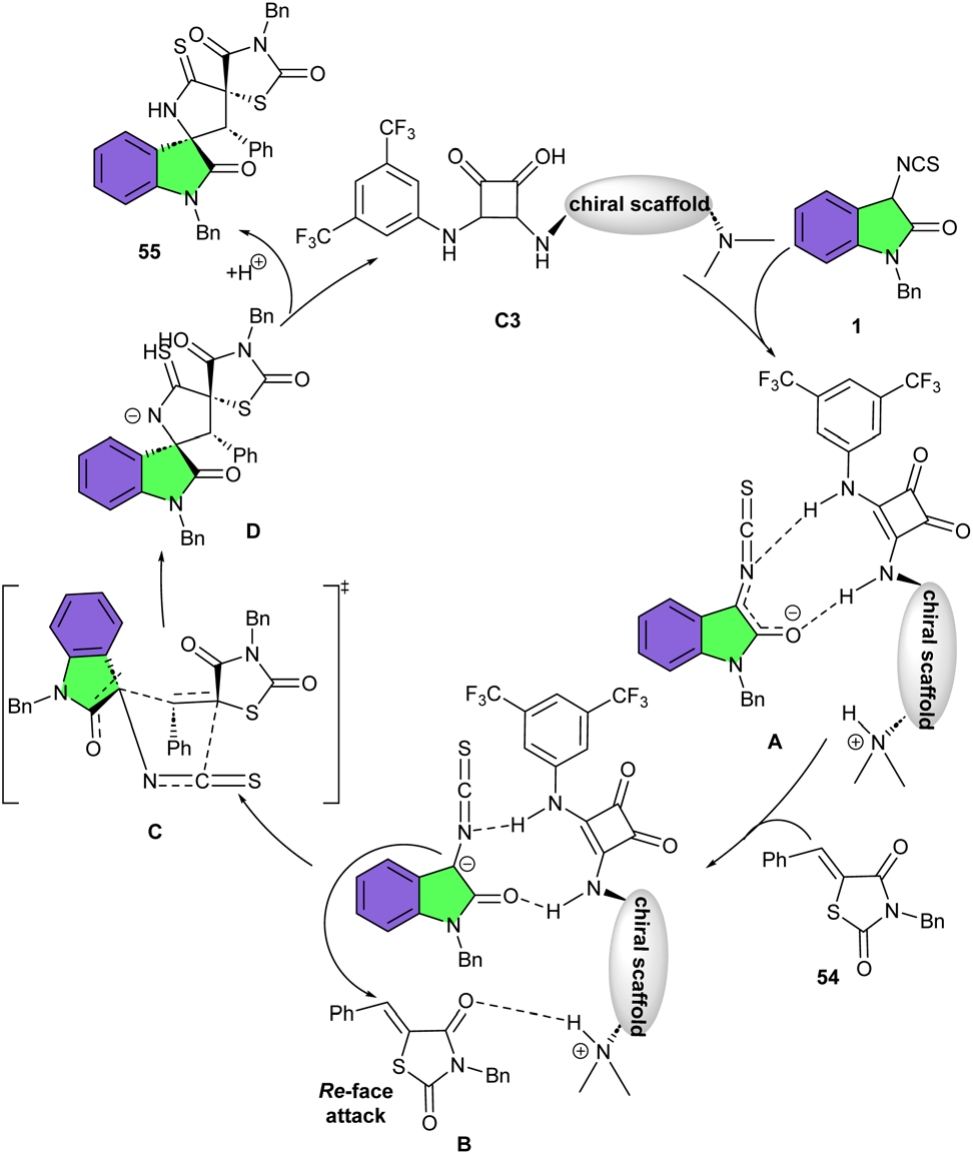
Rational mechanism for organocatalysis reaction of 3-isothiocyanato oxindoles and unsaturated thiazolidinones.

**Scheme 24:**
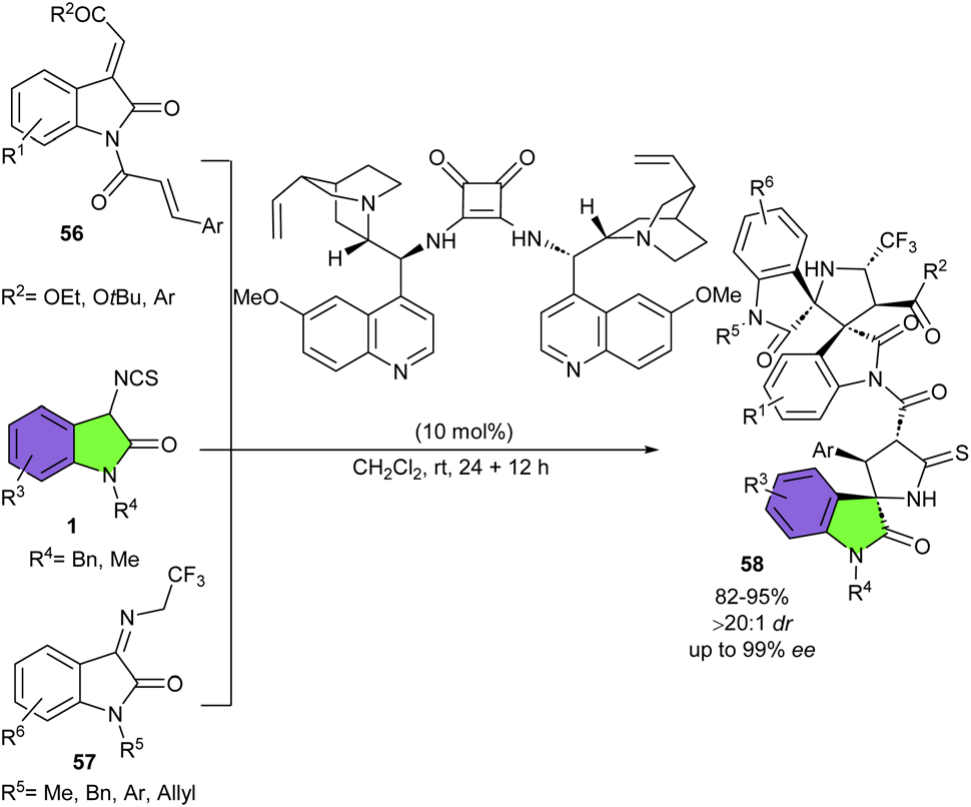
Organocatalysis reaction of 3-isothiocyanato oxindoles with cinnamoyl-3-ylideneoxindoles, and trifluoroethylisatin ketimines.

In 2018, Du and Song developed a strategy for the assembly of enantiopure oxindolepyrrolidone-thiazolidinone bispirocyclic compounds 55 (Scheme 22).^36^ For this purpose, they conducted the reaction of 3-isothiocyanato oxindoles 1 and unsaturated thiazolidinones 54 in the presence of various chiral bifunctional organocatalysts, involving cinchona-derived squaramides (I–VIII) and cinchona-derived thiourea IX. The spirocycles were obtained in high to excellent yields using catalysts I–VIII, but in moderate yield in the presence of IX. All catalysts led to exclusive diastereomer (98 : 2 to >99 : 1 dr), with high to excellent enantioselectivities (86–99% ee). Among them catalyst III was found to be the optimal catalyst, affording the products in high yields (up to 99%) with excellent diastereo- and enantioselectivities (up to >99 : 1 dr, >99% ee). As shown in Scheme 23, the tertiary amine unit of the quinine deprotonated the 3-isothiocyanato oxindole 1 to activate it by forming transition state A. The thiazolidinone 54 was activated by hydrogen bonding interaction with the protonated amine, which was then attacked by the activated 3-isothiocyanato oxindole from the Re-face *via* transition state B. Following intermolecular Michael addition/cyclization produced intermediate D through transition state C. Finally, D delivered spirocycle 55 along with the regeneration of the catalyst III after a protonation process. After a year, Du and Zhao introduced a one-step three-component Michael/Mannich–Michael/cyclization cascade reaction for the assembly of bispirooxindole-spirooxindoles 58 (Scheme 24).^37^ A bifunctional squaramide-catalyzed was employed in the reaction of 3-isothiocyanato oxindoles 1, cinnamoyl-3-ylideneoxindoles 56, and trifluoroethylisatin ketimines 57 to produce the corresponding spirocycles 58 with seven stereocenters in high yields (81–95%) with excellent stereoselectivities (up to >20 : 1 dr, 99% ee). This sequential cascade method could also be performed in a gram-scale reaction with the same excellent stereoselectivity (1.494 gr, 86%, >20 : 1 dr, 99% ee).

**Scheme 25:**
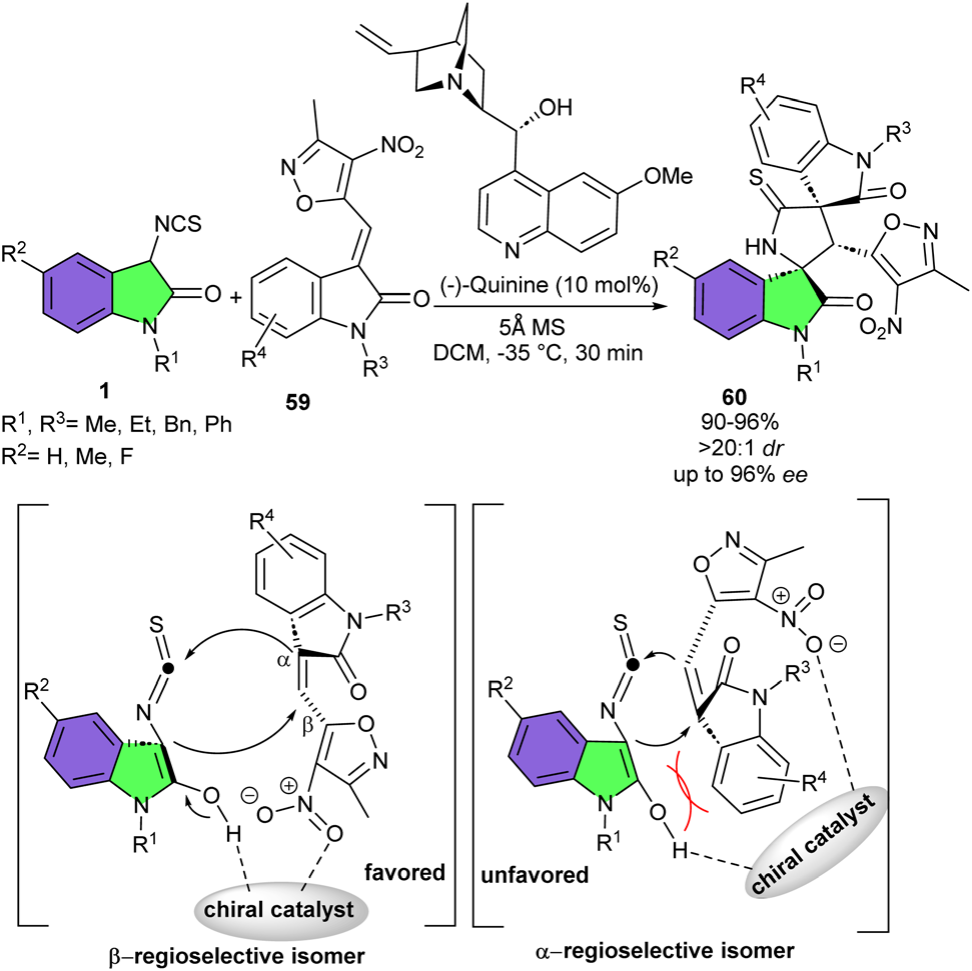
Organocatalysis reaction of 3-isothiocyanato oxindoles and 3-methyl-4-nitro-5-isatylidenyl-isoxazoles.

In 2019, Yuan and his team performed a novel stereo- and β-regioselective (3 + 2)-cycloaddition reaction of 3-isothiocyanato oxindoles 1 with 3-methyl-4-nitro-5-isatylidenyl-isoxazoles 59 for the synthesis of functionalized isoxazole-dispirobisoxindoles 60 (Scheme 25).^38^ The procedure carried out under the catalysis of commercially available quinine at −35 °C for 30 min. A wide spectrum of isoxazole-dispirobisoxindoles incorporating two different oxindole cores and three contiguous stereocenters were constructed in 90–96% yield, >20 : 1 dr and up to 96% ee. Various 3-isothiocyanato oxindoles with Et, Me, Ph and Bn substitutions on the N1-protecting, or bearing Me and F group on the phenyl ring, all were compatible. Additionally, the electronic and steric effects of functional groups on 3-methyl-4-nitro-5-isatylidenyl-isoxazoles had no impact on the reactivity and diastereoselectivity but had an obvious negative impact on the enantioselectivity. After assuming two possible transition state models, the authors noted that the formation of the α-regioselective isomer was not favorable due to the large steric hindrance resulting from the interaction of two oxindole moieties encountered in the first Michael addition step. Thus, it was more desirable for the quinine organocatalyst to facilitate β-regioselective annulation of 3-isothiocyanato oxindoles with 3-methyl-4-nitro-5-isatylidenyl-isoxazoles. In the favored transition state model, the nucleophilicity of the substrate 1 was enhanced after the interaction with the tertiary amine of quinine. Simultaneously, quinine formed a hydrogen bond with the nitro group of substrate 59. Thus, the Re face of the β-position of the electrophile 59 was attacked by the Si face of the enolate 1 under the control of the quinine catalyst by an efficient shielding effect, leading to the β-regioselective carbon anion. Eventually, the electron-poor isothiocyanate moiety was attacked by the Si face of intermediate, affording spirocycle 60 with (2′*R*,3′*S*,4′*R*)-configuration. The scale-up of the cycloaddition reaction led to the formation of the product in 0.53 gr, 94% yield, >20 : 1 dr, and 87% ee.

**Scheme 26:**
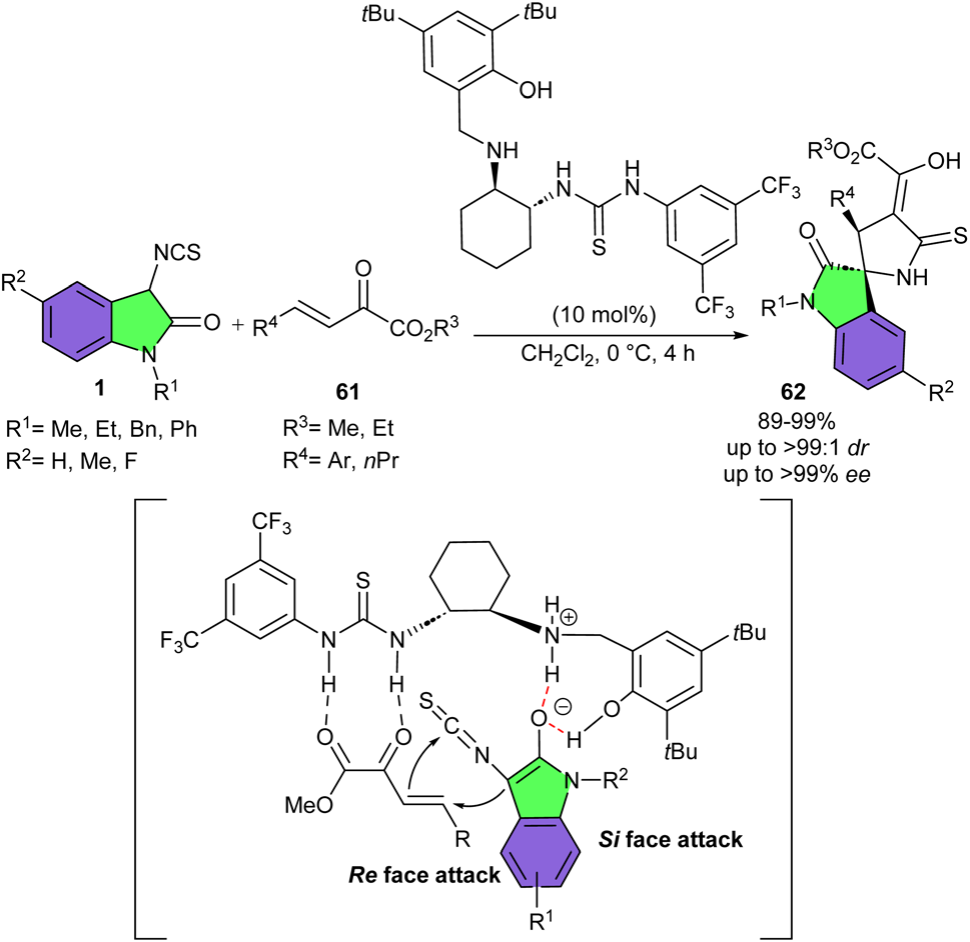
Thiourea-secondary amine catalyzed reaction of 3-isothiocyanato oxindoles and β,γ-unsaturated α-keto esters.

Thiourea-secondary amine catalyst was found to be an efficient catalyst for Michael/cyclization reaction of 3-isothiocyanato oxindoles 1 and β,γ-unsaturated α-keto esters 61 (Scheme 26).^39^ A wide variety of 2′-thioxospiro[indoline-3,4′-oxazolidin]-2-one complexes were constructed in high to excellent yields (89–99%) with excellent diastereo- and enantioselectivities (up to >99 : 1 dr, >99% ee). As shown in the transition state, 3-isothiocyanato oxindole was activated *via* deprotonation/enolization by the amine moiety of catalyst, generating the enolate. On the other hand, the thiourea moiety of catalyst activated β,γ-unsaturated α-keto ester through the double hydrogen bonding. Afterwards, the Si face of the enolate fixed by the double hydrogen-bonding with the ammonium salt and free OH group of the catalyst attacked the Re face of 61, and then the α-position of α-keto ester attacked the isothiocyanato to obtain spirooxindole 62. In should be noted that the multiple hydrogen bonds involved in the transition state model not only activate the two substrates but also play a crucial role in stereocontrol. Moreover, two derivatives of the spirocyclic products were found to behave good in anti-inflammatory activities, indicating potential chemotherapeutic property of these spirocycles.

**Scheme 27:**
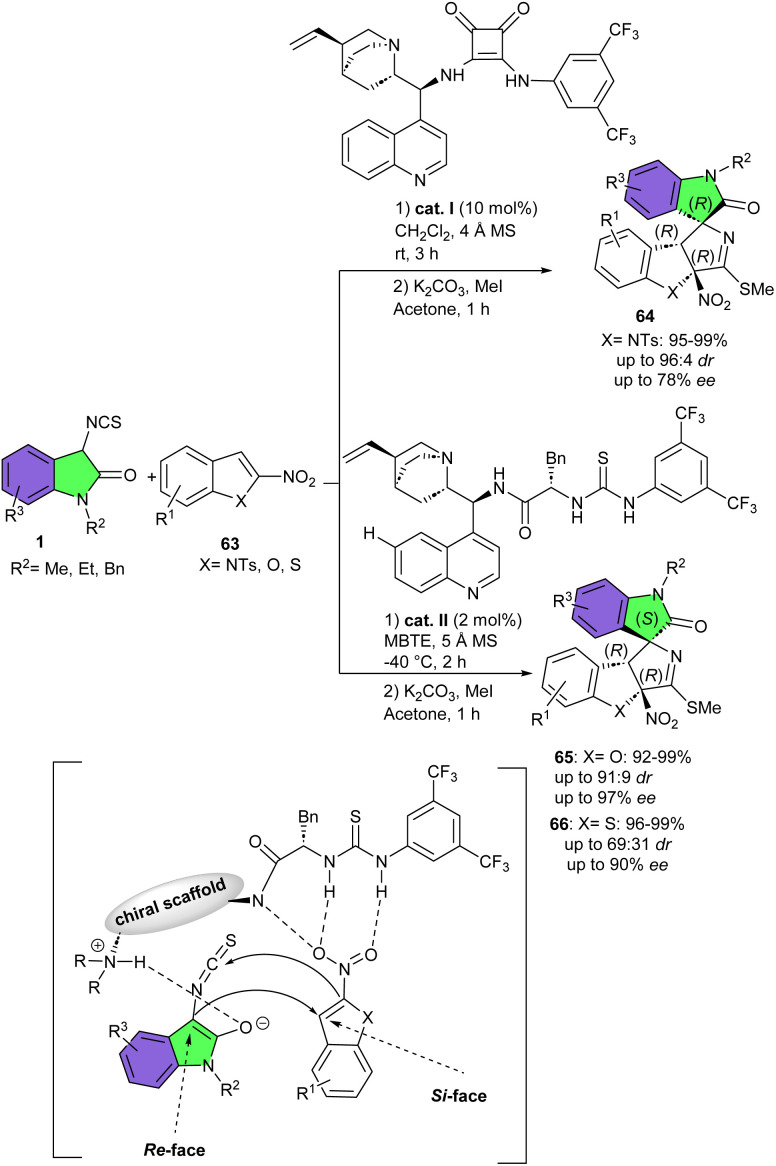
Organocatalysis reaction of 3-isothiocyanato oxindoles and 2-nitrobenzo heteroarenes.

In 2021, Yuan and co-workers used various 2-nitrobenzo heteroarenes 63 such as 2-nitrobenzofurans, 2-nitrobenzothiophenes and 2-nitroindoles as electrophiles in the reaction with 3-isothiocyanato oxindoles 1 (Scheme 27).^40^ Two bifunctional thiourea and bifunctional cinchonine-squaramide catalysts were chosen to achieve a diverse range of structurally diverse polycyclic spirooxindoles in high to excellent yields (92–99%) with high diastereo- and enantioselectivities (up to 94 : 6 dr, and 97% ee). Thiourea catalyst II was able to catalyze dearomative (3 + 2)-annulation reaction of 2-nitrobenzofurans, and 2-nitrobenzothiophenes with 3-isothiocyanato oxindoles, whereas cinchonine-squaramide I was an effective catalyst for the asymmetric annulation of 2-nitroindoles with 3-isothiocyanato oxindoles. Regardless of the electronic property and position of substitutions on the aryl ring of 3-nitroindoles or 3-isothiocyanato oxindole, all substrates displayed high reactivity. As displayed in the possible activation mode, electron-deficient 2-nitrobenzofuran or 2-nitrobenzothiophene was activated by the multiple hydrogen bonds of thiourea and amide motifs of the catalyst II. Simultaneously, 3-isothiocyanato oxindole was activated by the tertiary nitrogen of the cinchonidine of catalyst II to enhance the nucleophilicity of the C3-position of 3-isothiocyanato oxindole, to Re-face attack the Si-face of C3-position of 2-nitrobenzofuran or 2-nitrobenzothiophene. Then, intramolecular cyclization furnished polycyclic spirooxindoles with (C1*R*, C2*R*, C3*S*) configurations.

#### 2.1.3. Alkynes as electrophiles

**Scheme 28:**
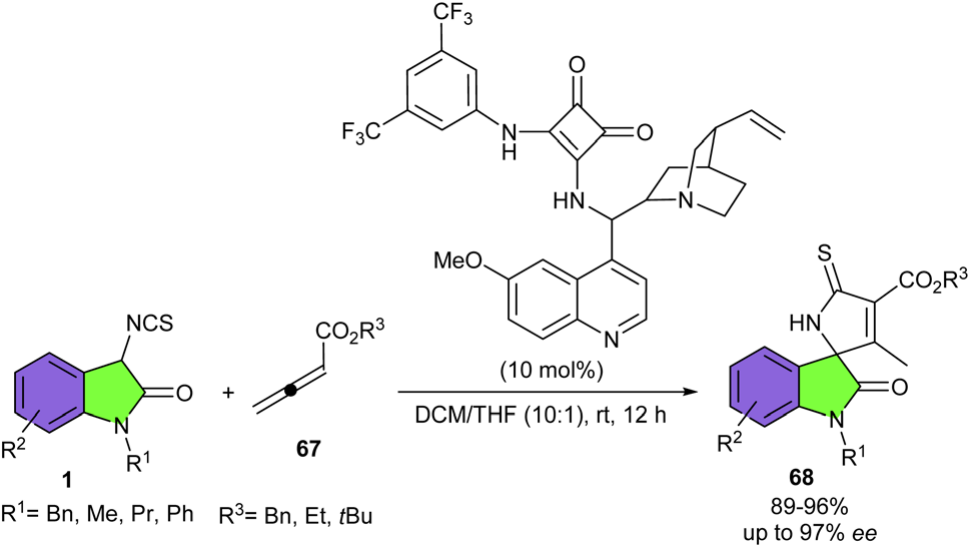
Cinchona alkaloid-derived organocatalyst catalyzed reaction of 3-isothiocyanato oxindoles and allenic esters or 2-butynedioic acid diesters.

**Scheme 29:**
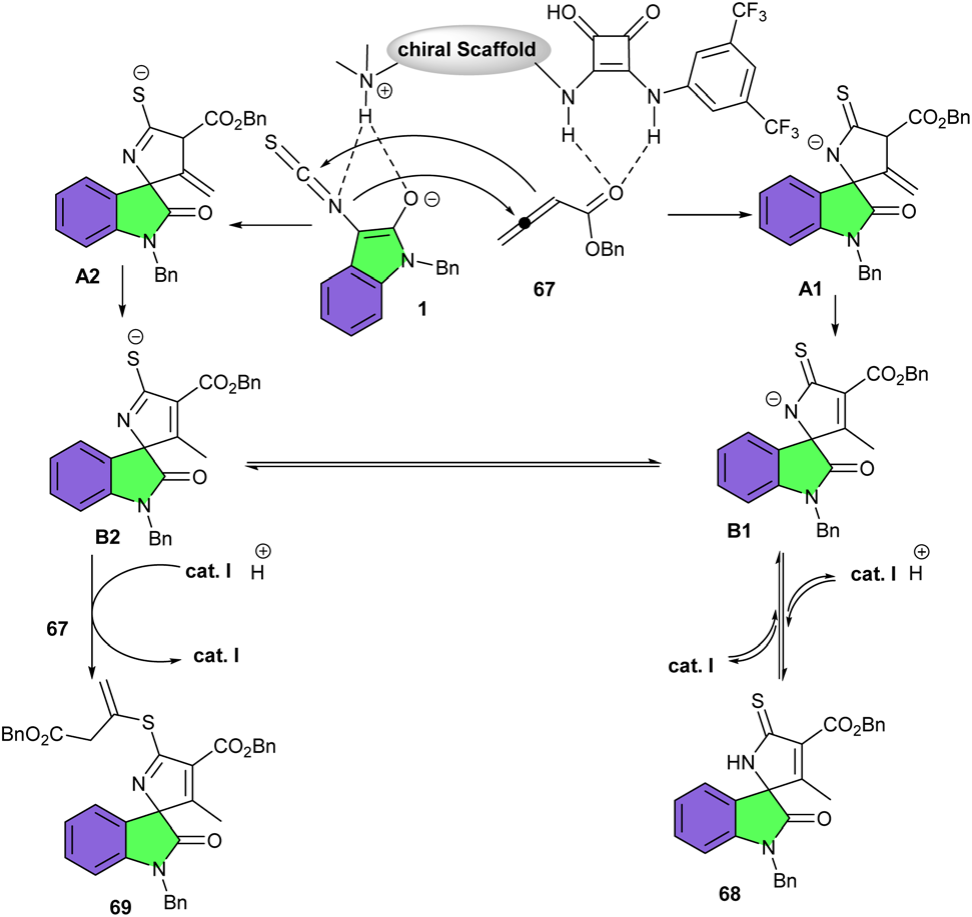
Credible mechanism for cinchona alkaloid-derived organocatalyst catalyzed reaction of 3-isothiocyanato oxindoles and allenic esters or 2-butynedioic acid diesters.

Shi introduced another novel cinchona alkaloid-derived organocatalyst for the asymmetric cycloaddition reaction between 3-isothiocyanato oxindoles 1 with allenic esters or 2-butynedioic acid diesters 67 (Scheme 28).^41^ A series of functionalized spirooxindoles 68 were obtained in high yields (89–96%) with high to excellent enantioselectivities (86–97%) under mild conditions. The authors could also synthesize different spirooxindole derivatives with the same high enantioselectivities *via* changing the ratio of the substrates. The reaction mechanism involved the interaction of the organocatalyst I with 67 through its strong hydrogen-bonding donor site and abstracted one proton from 1 with its amino base site. Subsequent intermolecular Michael addition/cyclization led to intermediate A1 or A2, and subsequent double bond migration generated intermediate B1 or B2. Intermediate B1 resulted in product 68 after a protonation step. While intermediate B2 was subjected to further intermolecular Michael addition with 67 and then protonation access to product 69 (Scheme 29).

#### 2.1.4. Imines as electrophiles

**Scheme 30:**
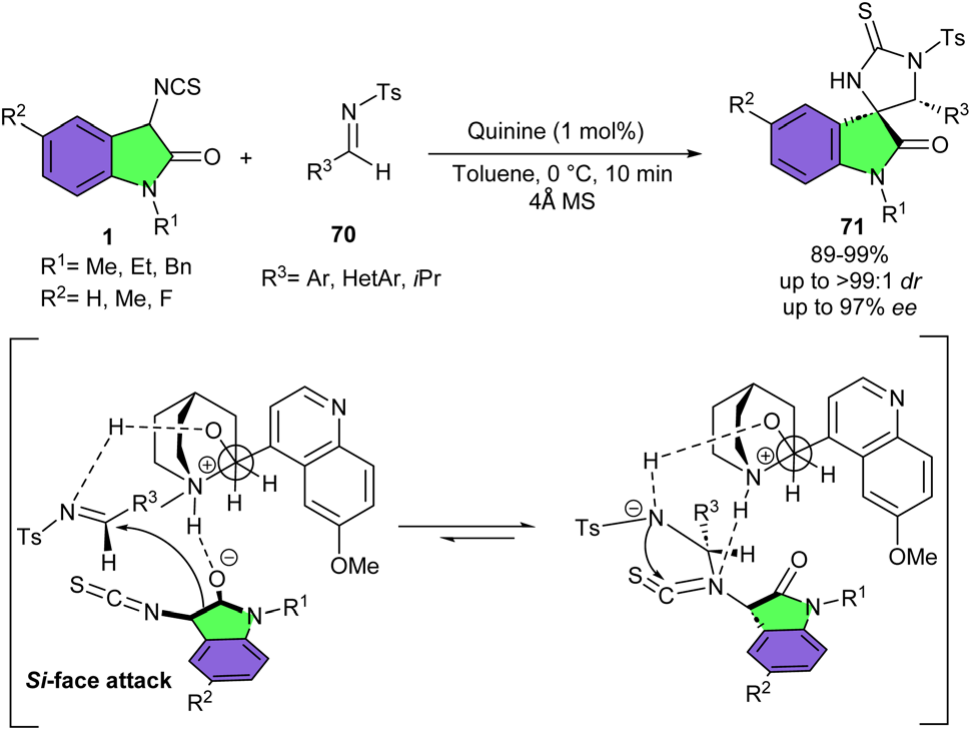
Quinine-catalyzed reaction of 3-isothiocyanato oxindoles and imines.

The enantioselective structurally diverse spiro[imidazolidine-2-thione-4,3′-oxindole] scaffolds 71 were obtained from quinine-catalyzed domino Mannich/cyclization reaction of 3-isothiocyanato oxindoles 1 with imines 70 (Scheme 30).^42^ The method had the advantages of commercially available catalyst, low catalyst loading (1 mol%), short reaction time, mild conditions, and high yields (up to 99%), and good to excellent diastereo- and enantioselectivity (up to >99 : 1 dr and up to 97% ee). A possible transition state was proposed for this asymmetric domino Mannich-cyclization process, in which C9–OH of quinine interacted with the nitrogen atom of tosyl-protected imine. Meantime, 3-isothiocyanato oxindole was enolized by deprotonation at its 3-position carbon atom by the tertiary amine of quinine. Then, the Si face of the enolate attacked the Si face of the imine. In the next step, the nitrogen anion nucleophilically attacked the electron-deficient C-atom of the isothiocyanato group of 1, delivering the product 71.

**Scheme 31:**
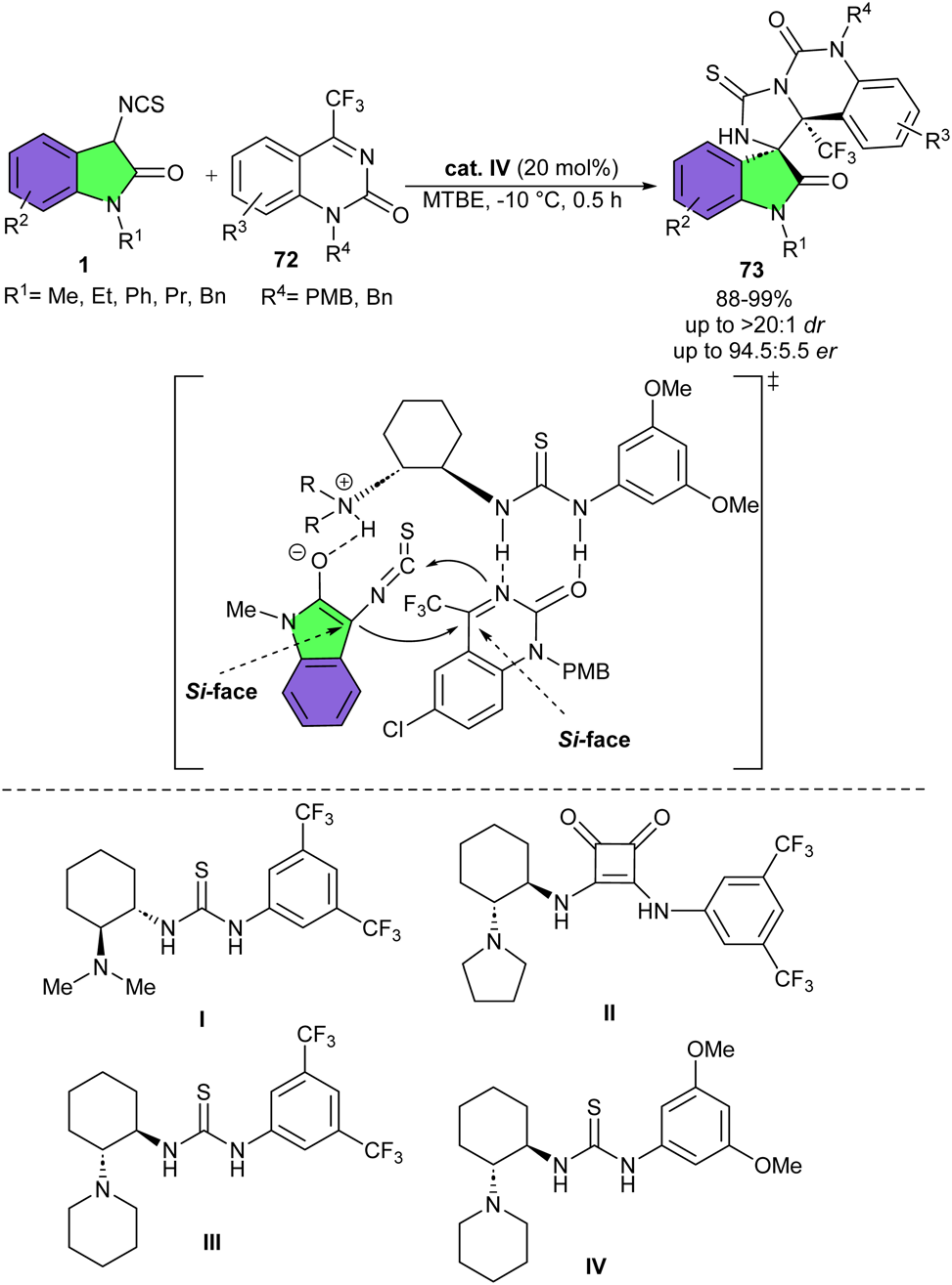
Cyclohexanediamine-derived bifunctional thiourea catalyzed reaction of 3-isothiocyanato oxindoles and cyclic ketimines.

Cyclohexanediamine-derived bifunctional thiourea IV can efficiently catalyze asymmetric Mannich/cyclization reaction between 3-isothiocyanato oxindoles 1 and various cyclic ketimines 72 (Scheme 31).^43^ Other organocatalysts I–III were not as effective as catalyst IV. In the procedure, 4-(trifluoromethyl)quinazolin-2-ones, saccharin derived cyclic ketimines, and benzo[1,4]oxazin-2-one derived cyclic ketimines as electrophiles reacted smoothly with 3-isothiocyanato oxindoles very under mild conditions, furnishing chiral polycyclic spiro-thioimidazolidine-oxindoles 73 in moderate to excellent yields (54–99%) with high diastereo- and enantioselectivities (up to >20 : 1 dr and 94.5 : 5.5 er). 3-Isothiocyanato oxindoles, bearing different alkyl, phenyl and benzyl substituents at the N1 position all reacted well, indicating changing the N1 group of 3-isothiocyanato oxindoles had no significant effect on the reactivity and stereoselectivity.

Also, electron-donating group was tolerated in the reaction, affording products high to excellent yields with acceptable enantioselectivities. For cyclic trifluoromethyl ketimines, the electronic nature of the substituents on the phenyl ring did not affect the yield and stereoselectivity. As shown in the transition state, the cyclic ketimine was activated and the hydrogen bonds of the thiourea of the catalyst, while the 3-isothiocyanato oxindole was enolized *via* deprotonation by the tertiary amine of IV. Under this dual-activation model, the enolate as the incoming nucleophile from the Si face attacked the Si face of cyclic ketimine, followed by the nucleophilic attack of the nitrogen anion on the electron-poor carbon atom of the isothiocyanato group, generating (*S*,*S*)-spiro-thioimidazolidine-oxindole.

**Scheme 32:**
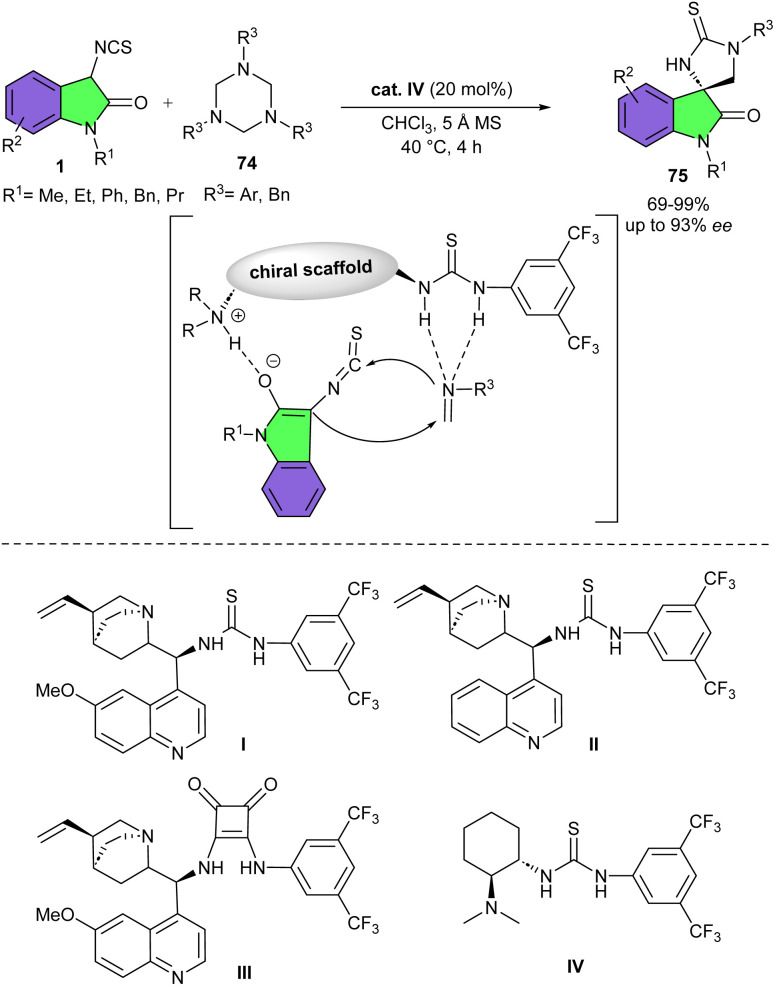
Organocatalysis reaction of 3-isothiocyanato oxindoles and 1,3,5-trisubstituted-hexahydro-1,3,5-triazines.

In another reaction, Yuan *et al.* utilized Takemoto's chiral bifunctional thiourea catalyst in the (3 + 2)-cycloaddition of 3-isothiocyanato oxindoles 1 with formaldimines 74 (Scheme 32).^44^ The study of four organocatalysts showed that the bifunctional chiral thiourea organocatalysts I and II derived from cinchona alkaloid produced the desired products in moderate yields and good stereoselectivities, while squaramide-cinchona alkaloid III resulted in a severe enantiomeric reduction. In this reaction, the less bulky chiral bifunctional thiourea IV provided spirocycle in better yield and enantiomeric excess. The authors studied the scope and limitation of this method by conducting the reactions of a variety of 3-isothiocyanato oxindoles containing different substituents on the nitrogen atom. The alkyl groups on the N1-position did not show influence on the yield and selectivity. While the Ph and Bn on the N1-position had negative effects in yield, enantioselectivity, respectively. Besides, electron-donating groups showed positive effects on the yield and enantioselectivity *versus* electron-withdrawing groups. On the other hand, the 1,3,5-trisubstituted-hexahydro-1,3,5-triazines containing different functional substituents on the aromatic rings were also compatible. The benzyl-substituted-triazine also led to the corresponding product in high yield but in a poor enantioselectivity presumably due to the smaller steric hindrance of the aliphatic than aromatic moieties. A Mannich/intramolecular cyclization process was suggested, initiated by the activation of formaldimines by double hydrogen bonding and deprotonation of 3-isothiocyanato oxindoles by the tertiary amine moiety of the catalyst. Then, under the stereocontrol of the chiral catalyst, the activated 3-isothiocyanato oxindole attacked the formaldimines from the Re face. Thus, the approach of the nitrogen anion of formaldimines to the –NCS group of 3-isothiocyanato oxindoles *via* an intramolecular annulation produced spiro-imidazolidinethione-oxindoles with *R* configuration. Finally, the synthetic utility of this method was demonstrated by the gram-scale synthesis of the product (1.10 gr, 96%, 93% ee).

#### 2.1.5. Azides as electrophiles

**Scheme 33:**
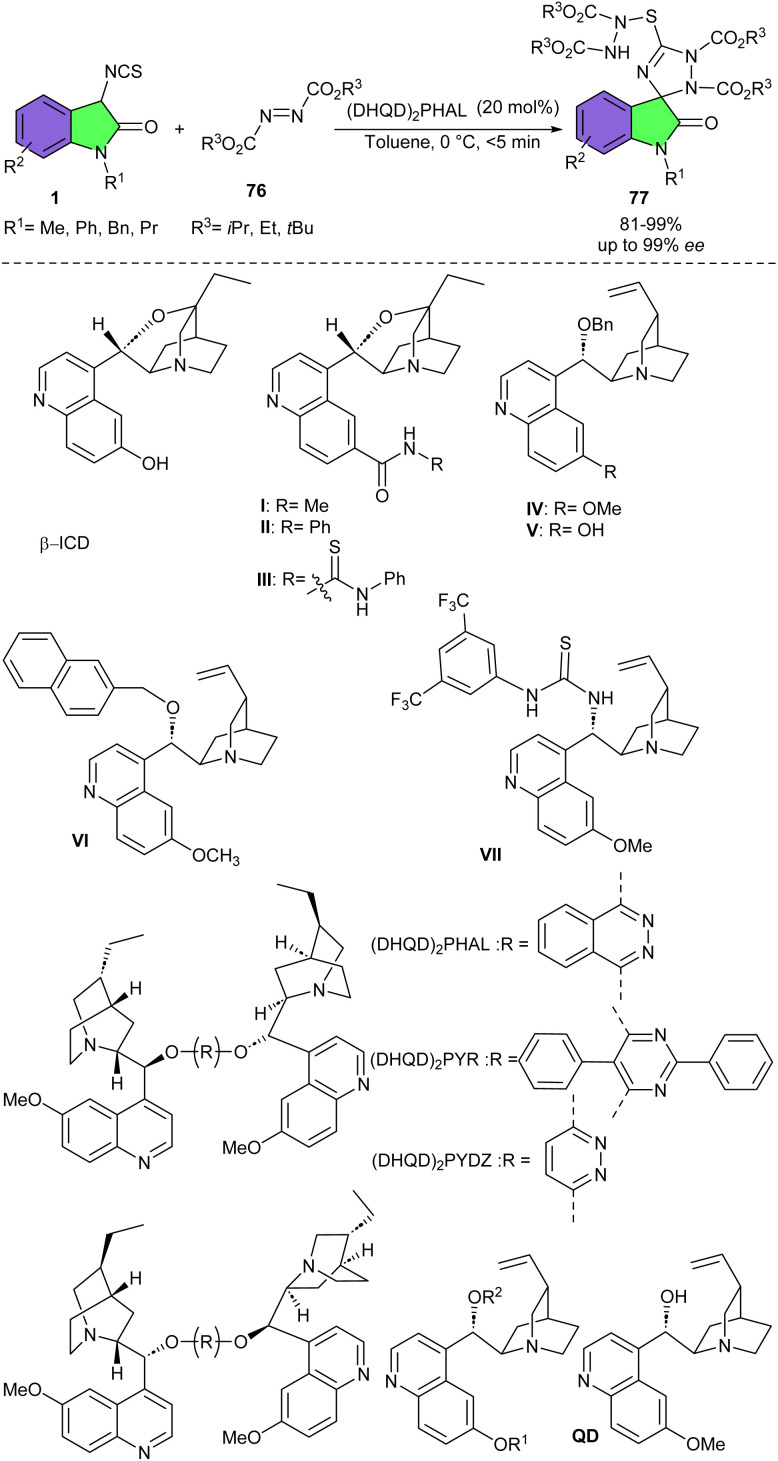
(DHQD)_2_PHAL-catalyzed reactions of 3-isothiocyanato oxindoles and azodicarboxylates.

Among the screening of various organocatalysts, Shi and his co-workers found that only (DHQD)_2_PHAL could catalyze the (3 + 2)-cycloaddition between 3-isothiocyanato oxindoles 1 and azodicarboxylates 76 with high enantioselectivity, while other catalysts had no stereoselectivity (β-ICD and cat. 1–7) or moderate enantioselectivity ((DHQD)_2_PYDZ and QD) (Scheme 33).^45^ A wide range of spirooxindoles were well synthesized only within 5 minutes. Moreover, a new spirooxindole derived from the reaction of 3-isothiocyanato oxindole with two molecules of azodicarboxylate was also constructed in good yield (75%) with the same high enantioselectivity (95% ee) under these standard conditions. The authors also studied the biological activities of two derivatives of these obtained spirooxindoles, which exhibited acceptable biological activity.

### 2.2. Metal catalytic (3 + 2)-cyclization reactions of 3-isothiocyanato oxindoles

#### 2.2.1. Strontium catalysis

**Scheme 34:**
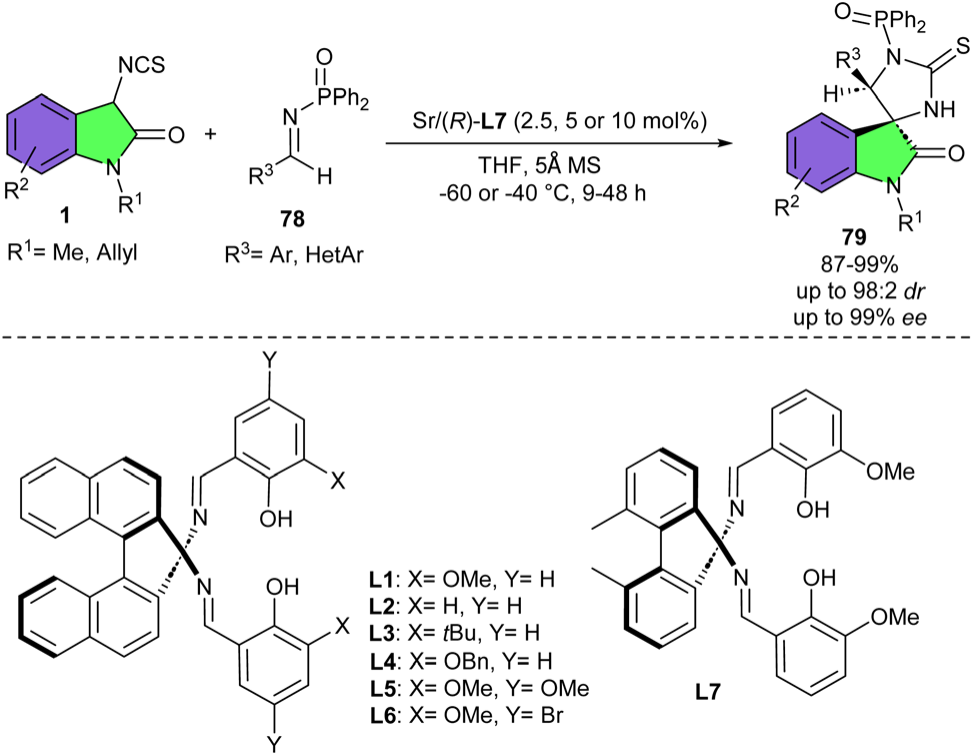
Sr-catalyzed Mannich/cyclization reaction of 3-isothiocyanato oxindoles and imines.

In 2012, Kanai and co-workers reported Sr-catalyzed asymmetric Mannich/cyclization cascade reaction of 3-isothiocyanato oxindoles 1 with imines 78 (Scheme 34).^13^ They investigated reactivity of various metal catalysts, including Bu_2_Mg, Ca(OiPr)_2_, Sr(OiPr)_2_, Ba(OiPr)_2_, Al(OiPr)_3_, Ni(OAc)_2_, wherein Sr(OiPr)_2_ showed the best result. In the next step, to improve the stereoselectivity of the reaction, several Schiff bases L1–L7 were tested. Lack an *ortho*-OMe substituent in ligands L1–L4 resulted in poor enantioselectivity. Electronic tuning of Ligands L5 and L6 also led to moderate selectivity. While Schiff base L7 having a 2,2′-biphenyldiamine backbone, and two *ortho*-OMe substituents proved to be the best ligand for Sr(OiPr)_2_, affording the desired product in 95% yield with excellent stereoselectivity (91 : 9 dr, 93% ee). The protocol offered a direct access to enantioenriched spiro[imidazolidine-4,3′-oxindole] compounds. Generally, 3-isothiocyanato oxindoles having *N*-allyl protected, Schiff base demonstrated better reactivity and stereoselectivity. For, N–Me oxindole, the product was obtained in good yield and stereoselectivity. In the case of imines, substituents at the *para*-, *meta*-, and *ortho*-positions on the aryl ring all were well tolerated. Even for the less reactive 4-MeO-substituted imine, Schiff base with the binaphthyl backbone showed better reactivity, and product was achieved in 92% yield and 93% ee, albeit with modest diastereoselectivity. Imines bearing electron-withdrawing groups also resulted in high selectivity.

#### 2.2.2. Zinc catalysis

**Scheme 35:**
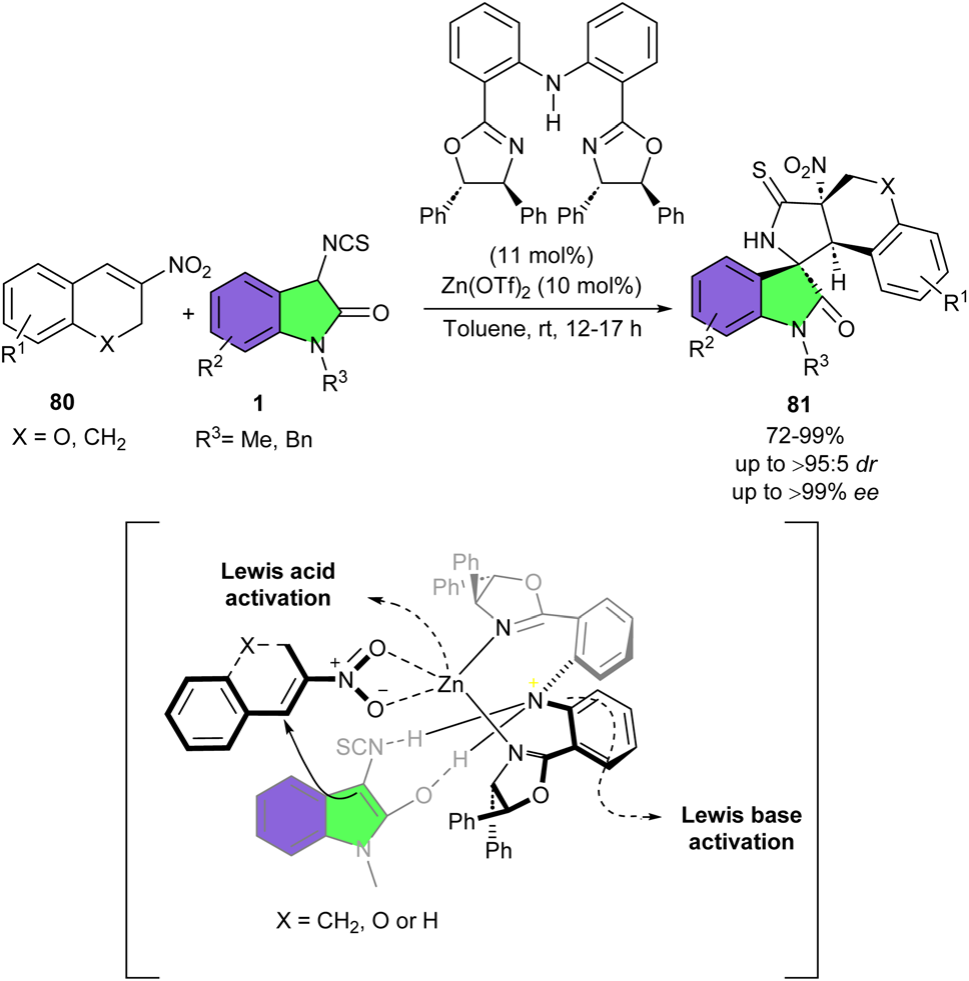
Zn-catalyzed Michael/cyclization reaction of 3-isothiocyanato oxindoles and 3-nitro-2*H*-chromenes.

In 2014, the first Zn(OTf)_2_-catalyzed asymmetric Michael/cyclization cascade reaction of 3-isothiocyanato oxindoles 1 with 3-nitro-2*H*-chromenes 80 was developed by Chen *et al.* (Scheme 35).^46^ Various polycyclic spirooxindoles 81 having 1,3-nonadjacent tetrasubstituted carbon stereocenters were constructed in good to excellent yield (72–99%) with excellent distereo- and enantioselectivities (up to >95 : 5 dr, and >99% ee). The use of a chiral bis(oxazoline) ligand was found to be necessary due to the important role of the free NH moiety in controlling the stereochemistry. The substrate scope showed good tolerance respect to 3-nitro-2*H*-chromenes and 3-isothiocyanato oxindoles and many functional groups were compatible. When the oxygen atom of 3-nitro-2*H*-chromene was replaced by a methylene group, the reaction also proceeded well, providing the desired product in 95% yield with >95 : 5 dr and >99% ee. Additionally, this synthetic method could be carried out in the gram scale, yielding 1.02 gr, 67% yield of product, >95 : 5 dr, >99% ee. As seen in the transition state model, chiral Zn(OTf)_2_/bis(oxazoline) complex formed a bifunctional activation mode, wherein the Zn(ii) moiety acted as a Lewis acid to activate the electron-deficient alkene through the coordination between Zn(ii) and the nitro group. On the other hand, the N-atom of the free NH group acted as a Lewis base to nucleophilically attacked from the Re face of alkene 80 through a hydrogen-bonding interaction with 3-isothiocyanato oxindole 1.

**Scheme 36:**
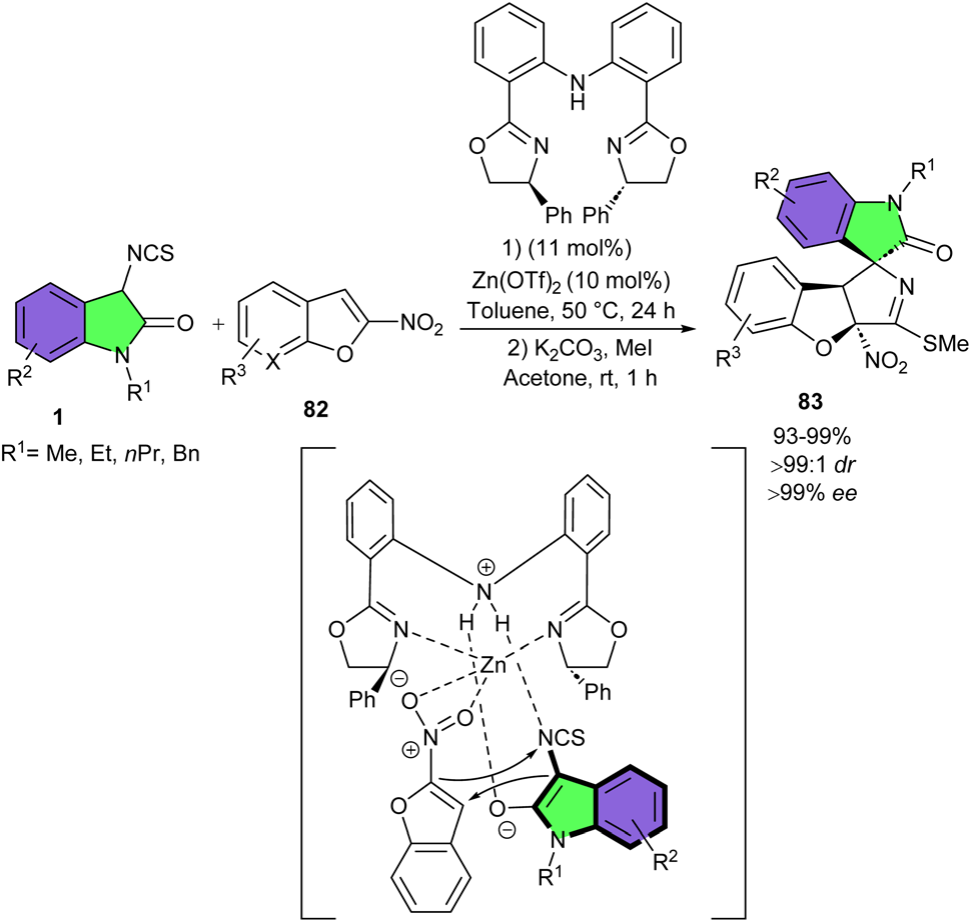
Zn-catalyzed (3 + 2)-cyclization reaction of 3-isothiocyanato oxindoles and 2-nitrobenzofurans.

**Scheme 37:**
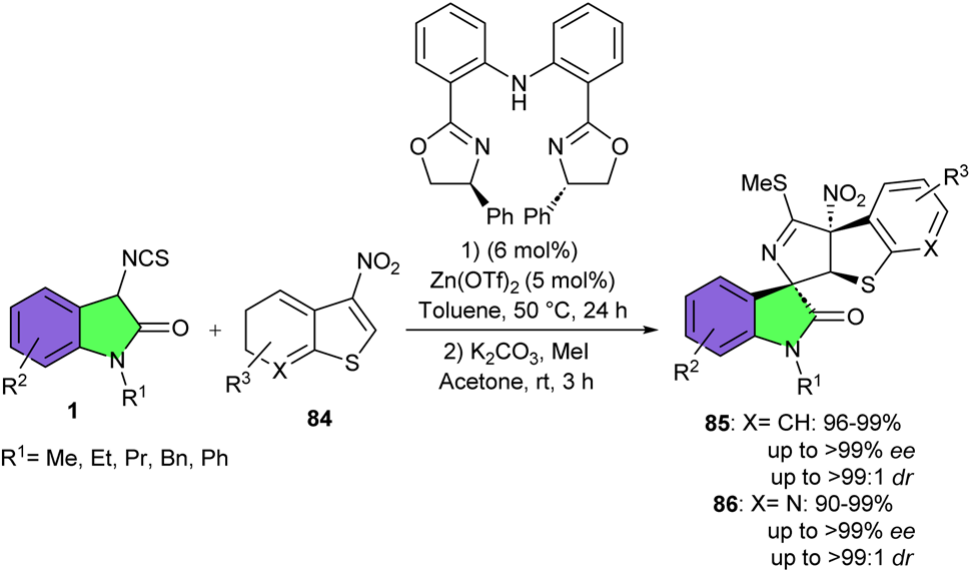
Zn-catalyzed (3 + 2)-cyclization reaction of 3-isothiocyanato oxindoles with 3-nitrobenzothiophenes or 3-nitrothieno[2,3-*b*]pyridine.

In 2018, Yuan and his team utilized the same Zn(OTf)_2_/bis(oxazoline) catalytic system for the asymmetric dearomative (3 + 2)-cycloaddition reaction of 3-isothiocyanato oxindoles 1 with 2-nitrobenzofurans 82 (Scheme 36).^47^ They could synthesize a series of spirooxindoles 83 containing a 2,3-dihydrobenzofuran motif and three contiguous stereocenters in high to excellent yields (93–99%), with excellent diastereo- and enantioselectivities (up to >99 : 1 dr, and >99% ee). 3-Isothiocyanato oxindoles, bearing different substituents such as Et, Pr, and Bn at the N1-position displayed high reactivities. Moreover, the presence of either electron-withdrawing or electron-donating group on the C5- or C7-position of the aromatic ring were also well tolerated. Regardless of the electronic property, bulkiness, or position of substituents on the phenyl ring of 2-nitrobenzofurans, all provided the corresponding products in very high yield and selectivity. Although 3-substituted 2-nitrobenzofuran derivatives were not workable in this synthetic method, while the reaction of methyl 5-nitrofuran-2-carboxylate with 3-isothiocyanato oxindole gave unsatisfactory results (41% yield, 62 : 38 dr and 0.27 ee). The suitability of this method was then showed by the gram-scale synthesis of the product by using 1 mmol scale of the reactants, yielding spirocyclic product in 90% yield, with >99 : 1 dr, and >99% ee. Based on preliminary mechanistic investigations, a plausible transition state model was proposed in which a Zn(OTf)2/L complex was formed. This zinc complex acted as Lewis acid and activated 2-nitrobenzofurans by coordinating of Zn(ii) with the nitro group. 3-Isothiocyanato oxindole was directed by the NH group to serve as a Lewis base to nucleophilically attack the Re face of C3-position of 2-nitrobenzofurans under the bifunctional activation of the Zn(OTf)_2_/L complex. The subsequent intramolecular annulation from the C2-position of 2-nitrobenzofuran to the –NCS group delivered the product 83 with stereospecific configuration. The same research group used this zinc catalysis system for another (3 + 2)-cycloaddition reactions of 3-isothiocyanato oxindoles 1 with 3-nitrobenzothiophenes or 3-nitrothieno[2,3-*b*]pyridine 84 (Scheme 37).^48^ A wide spectrum of structurally diverse spirooxindole scaffolds 85, 86 containing three contiguous stereocenters were obtained in quantitative yields with excellent diastereo- and enantioselectivities. The use of a chiral tridentate nitrogen ligand had an important role in achieving high enantioselectivity. All substrates were well tolerated in this stereoselective transformation, with yields, diastereomeric ratios, and enantiomeric excesses showing very little sensitivity to electronic and steric changes. However, the dearomative (3 + 2)-cycloaddition reactions of 2-methyl substituted 3-nitrobenzothiophene and 3-nitrothiophene with of 3-isothiocyanato oxindole did not work, indicating significantly lower reactivity of these two reactants compared to 3-nitrobenzothiophenes. The method was also successful for the large-scale synthesis of the product without loss of stereoselectivity (1.204 gr, 99% yield, >99% ee, >99 : 1 dr).

#### 2.2.3. Magnesium catalysis

**Scheme 38:**
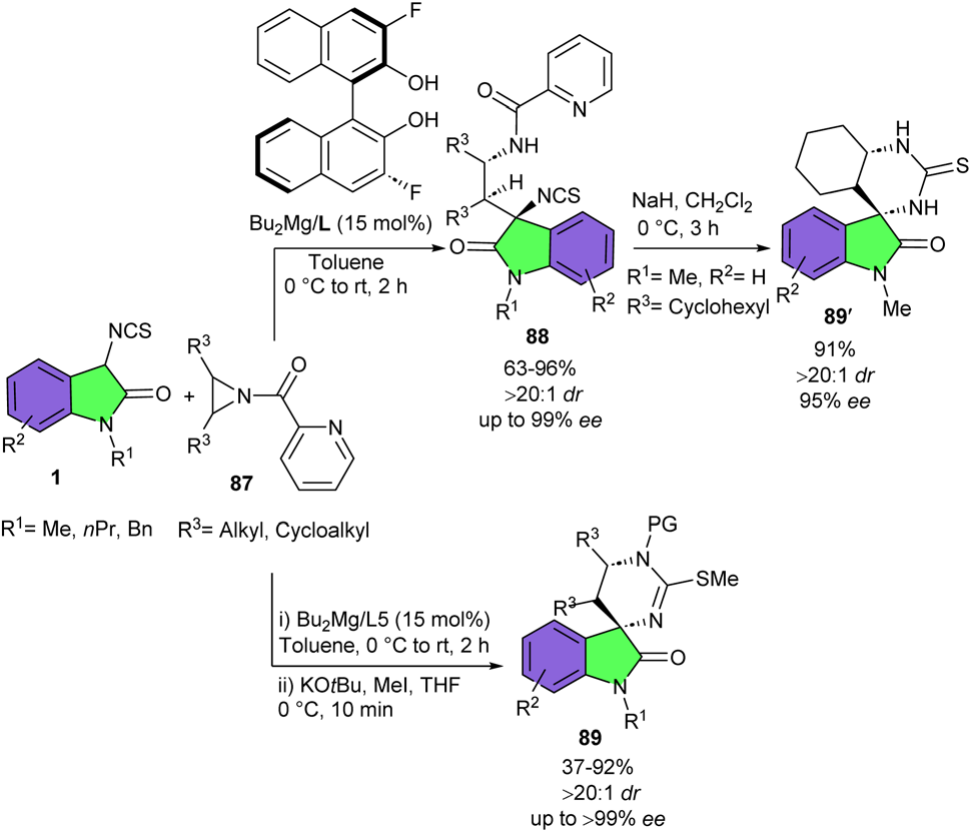
Mg-catalyzed (3 + 3)-cyclization reaction of 3-isothiocyanato oxindoles and aziridines.

**Scheme 39:**
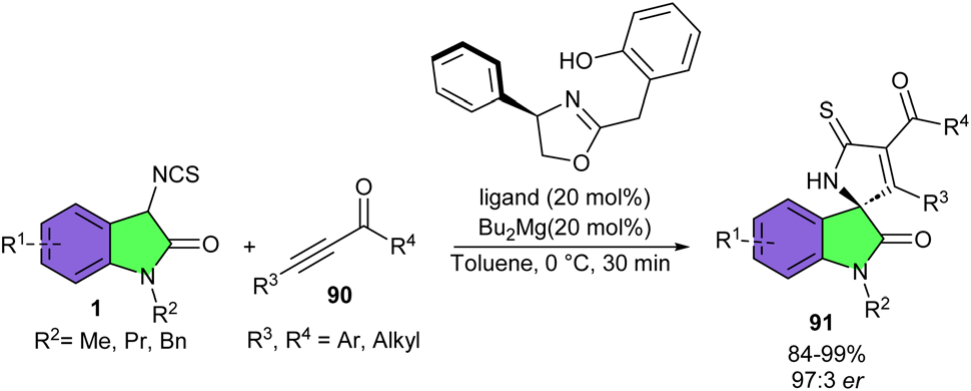
Mg-catalyzed (3 + 2)-cyclization reaction of 3-isothiocyanato oxindoles and alkynyl ketones.

**Scheme 40:**
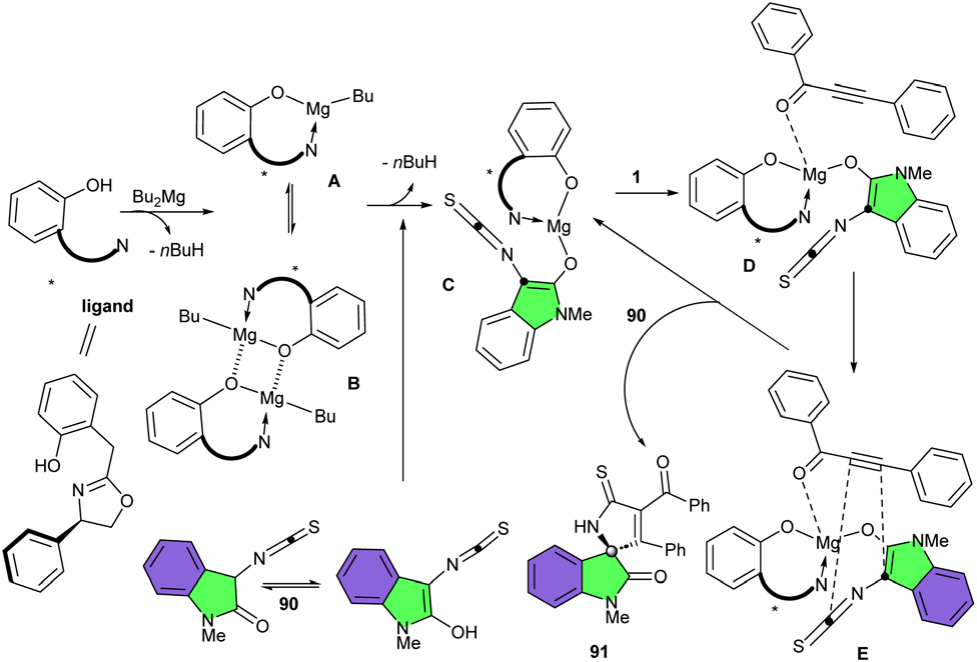
Possible mechanism for Mg-catalyzed (3 + 2)-cyclization reaction of 3-isothiocyanato oxindoles and alkynyl ketones.

Wang and his colleagues were able to achieve two different products by changing conditions in the reaction of 3-Isothiocyanato oxindoles 1 and aziridines 87 (Scheme 38).^49^ When 3-Isothiocyanato oxindoles reacted with aziridines in the presence of Bu_2_Mg as a catalyst and (*R*)-3,3′-fluorous-BINOL as a chiral ligand, ring-opening of aziridines took place to form adduct 88. This enantioenriched ring-opened product by having a free isothiocyano group could be easily coupled with the amino group of amino acids and peptides to modify their structure. Additionally, the ring-opened product was also utilized for the preparation of a new bifunctional amine-thioureas organocatalyst bearing multiple hydrogen-bonding donors. In the next step, the authors treated this ring-opened product in the presence of NaH in CH_2_Cl_2_ at 0 °C to obtain the (3 + 3)-cycloaddition product 89′. On the other hand, treating 3-Isothiocyanato oxindoles and aziridines in the presence of Bu_2_Mg and ligand, and then adding KO*t*Bu and MeI to the reaction mixture, could be resulted in the formation of spirocyclic frameworks 89 in 37–92% yield, >20 : 1 dr, and up to >99% ee. The Wang's group again employed Bu_2_Mg as the catalyst with a oxazoline-OH type chiral ligand derived from *ortho*-hydroxyphenylacetic acid, in the (3 + 2)-cycloaddition reaction of 3-isothiocyanato oxindoles 1 and alkynyl ketones 90 (Scheme 39).^50^ A series of enantiopure spirooxindoles were well synthesized in good to excellent yields (83–99%) with good enantioselectivities (up to 97 : 3 er). 3-Isothiocyanato oxindoles having N–Me, N-Bn, and N–Pr protected group reacted smoothly with aromatic enones bearing substituents at *para*-, *meta*-, and *ortho*-positions on the aromatic ring or heteroaryl enones. As shown in the plausible mechanism, 90 was coordinated to the magnesium center to bring about the formal (3 + 2)-cycloaddition process. Next, another molecule of 3-isothiocyanato oxindoles 1 participated in this process to allow the cyclization product 91 to liberate and regenerate the pre-catalyst for the next catalytic cycle (Scheme 40).

**Scheme 41:**
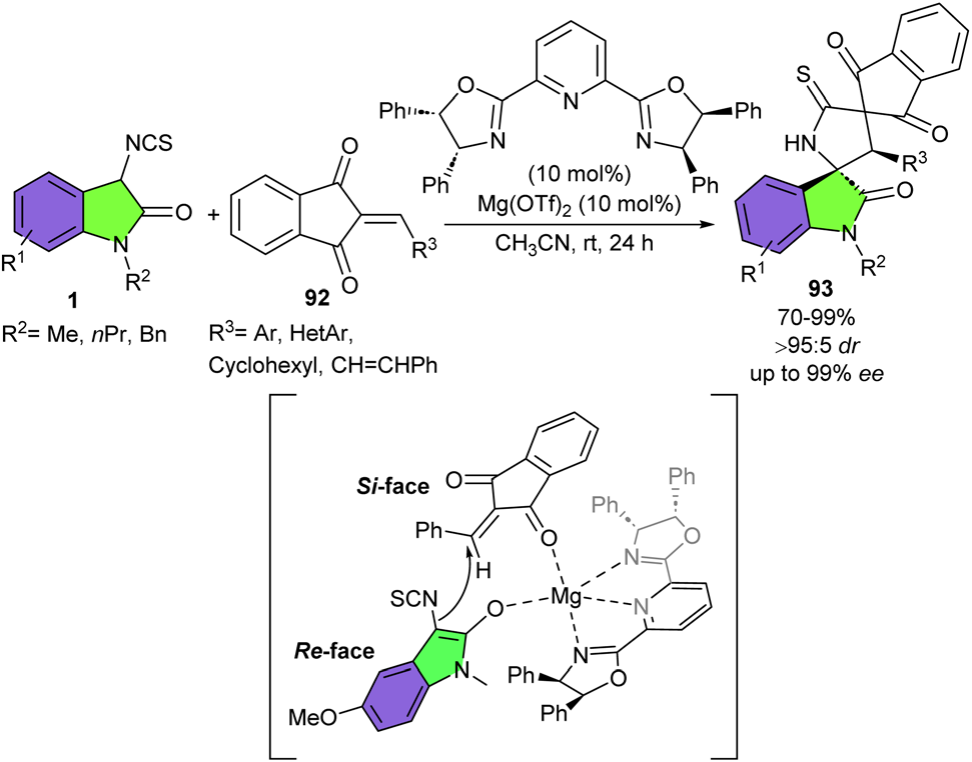
Mg-catalyzed Michael/cyclization reaction of 3-isothiocyanato oxindoles and 2-arylidene-1,3-indanediones.

In 2025, Tan *et al.* developed an asymmetric Michael/cyclization cascade reaction between 3-isothiocyanato oxindoles 1 and 2-arylidene-1,3-indanediones 92 (Scheme 41).^51^ For this purpose, they used a magnesium catalyst in the combination with a chiral tridentate oxazoline ligand, to provide bispiro[indanedione-oxindole-pyrrolidinyl] scaffolds 93 in good to excellent yields (70–99%) with good diastereo- and enantioselectivities (up to >95 : 5 dr, and 99% ee). 3-Isothiocyanato oxindoles containing both electron-withdrawing and -donating groups at different positions on the aryl ring as well as 3-isothiocyanato oxindoles with different protecting groups, such as *N*-methyl, *N*-benzyl and *N*-propyl showed good compatibility in this metal catalysis reaction. The researchers suggested a possible activation mode in which Mg(OTf)2 not only acts as a Lewis acid to activate 2-arylidene-1,3-indanedione *via* coordination of Mg(ii) to the carbonyl group but also played as a Lewis base to activate 3-isothiocyanato oxindole. Subsequently, the Re face of the activated 3-isothiocyanato oxindole 1 attacked the Si face of 2-arylidene-1,3-indanedione 92, followed by intramolecular cycloaddition towards the target product 93. The synthetic application of this method was demonstrated by the gram-scale synthesis of the product (1.72 gr, 78%, 95 : 5 dr, 86% ee), and further transformations of the spirocycles. Furthermore, evaluation of spirocycles revealed that one of the derivatives could exhibit promising antitumor activity against two human cancer cell lines.

## 3. Conclusions

As discussed in this context, both organocatalysis and metal catalysis systems can be well applied to various aldol/cyclization, Mannich/cyclization, Michael/cyclization, (3 + *n*) cyclization and self-cyclization/addition reactions involving 3-isothiocyanato oxindoles. Both reaction systems benefit from low temperatures and mild conditions. However, due to the bulky and specific structure of organocatalysts, the efficiency and selectivity are more obvious in organocatalysis systems than in metal catalysis. In most cases, increasing the volume of the chiral portion of organocatalyst can lead to a significant increase in stereoselectivity. In the case of metal catalysis reactions, the use of a chiral ligand is found to be essential to achieve high enantioselectivity.

Spirocyclic ring systems are promising as medicinally relevant compounds in drug discovery. Since 3-isothiocyanato oxindole is a good and potent candidate for the synthesis of novel complex spirocyclic scaffolds, the development of efficient and practical catalytic systems is highly desirable. It is worth noting that the design and development of highly diastereoselective and enantioselective methods for the synthesis of polyfunctionalized spirooxindoles containing one or more heteroatoms at their C3-position is still in demand. This need is especially felt in the field of medicinal chemistry and drug discovery. In this regard, the use of greener and more sustainable approaches such as photochemistry or electrochemistry as alternatives for metal catalysis could be attractive. Furthermore, the use of simple, commercially available chiral ligands in combination with metal catalysts can be beneficial to provide excellent stereoselective conditions.

## Conflicts of interest

There are no conflicts to declare.

## Data Availability

No primary research results, software or code have been included and no new data were generated or analysed as part of this review.
